# *UGT1A1* genotype testing for irinotecan: A guideline developed by the UK Centre of Excellence in Regulatory Science and Innovation in Pharmacogenomics (CERSI‐PGx)

**DOI:** 10.1002/bcp.70659

**Published:** 2026-07-02

**Authors:** Dharmisha Chauhan, Cinzia Dello Russo, Jack Thompson, Dyfrig A. Hughes, Katherine Payne, Maria Tsakiroglou, Paul Ross, Rena Chauhan, Simon Jenkinson, Jessica Keen, Sophie Harding, Eleanor Lau, Michael Braun, Roshan Agarwal, Richard Adams, Elisabeth Rolf, Farah Al‐Sheikhli, Sarah Mitchell, Ruth Hammond, Rosie Roberts, Lorna Cairns, Shirley Heggarty, Janet Graham, Justin Mencel, Munir Pirmohamed

**Affiliations:** ^1^ North Thames Genomic Medicine Service Alliance, Great Ormond Street Hospital for Children NHS Foundation Trust London UK; ^2^ Royal Marsden NHS Foundation Trust London UK; ^3^ Department of Pharmacology and Therapeutics, Institute of Systems, Molecular and Integrative Biology University of Liverpool Liverpool UK; ^4^ Department of Translational Medicine and Surgery, Section of Pharmacology Università Cattolica del Sacro Cuore—Fondazione Policlinico Universitario A. Gemelli, IRCCS Rome Italy; ^5^ Guy's and St Thomas' NHS Foundation Trust London UK; ^6^ Centre for Health Economics and Medicines Evaluation North Wales Medical School Bangor University Bangor UK; ^7^ Centre for Health Economics Faculty of Biology University of Manchester Manchester UK; ^8^ Royal Free London NHS Foundation Trust London UK; ^9^ Christie NHS Foundation Trust Manchester UK; ^10^ Division of Evolution, Infection and Genomics University of Manchester Manchester UK; ^11^ University Hospital of Wales, Cardiff and Vale University Health Board Cardiff UK; ^12^ Swansea Bay University Health Board Swansea UK; ^13^ University Hospitals of Northamptonshire Northampton UK; ^14^ Velindre Cancer Centre Velindre University NHS Trust Cardiff UK; ^15^ Cardiff University Cardiff UK; ^16^ Dorset County Hospital NHS Foundation Trust, Dorchester Dorset UK; ^17^ Mount Alvernia Hospital Guildford UK; ^18^ Western Health and Social Care Trust Londonderry UK; ^19^ Belfast Health and Social Care Trust Belfast UK; ^20^ West of Scotland Cancer Network Glasgow UK; ^21^ Wolfson Centre for Personalised Medicine, Centre for Drug Safety Science University of Liverpool Liverpool UK

**Keywords:** diarrhoea, irinotecan, neutropenia, pharmacogenetic testing, UGT1A1

## Abstract

Irinotecan, a topoisomerase I inhibitor, is available as both non‐pegylated and pegylated formulations. The non‐pegylated formulation is licensed for use in advanced colorectal cancer either in combination with other agents or as monotherapy. However, it is also used off‐label across a range of gastrointestinal malignancies and in rare malignancies such as glioblastoma and sarcomas. The pegylated formulation is licensed for use as combination therapy in adult patients with metastatic pancreatic adenocarcinoma. Irinotecan is hydrolysed to its active metabolite, SN‐38, which is predominantly inactivated by the enzyme uridine diphosphate glucuronosyltransferase UGT1A1. UGT1A1 is encoded by the gene *UGT1A1*, which is polymorphically expressed, with allele frequencies varying across populations. Poor metabolizers carry two variants that reduce UGT1A1 enzyme expression or activity, leading to increased risk of irinotecan toxicity. Any patient who is about to be prescribed irinotecan for an epithelial malignancy should have pharmacogenetic testing, to identify clinically relevant *UGT1A1* variants, where testing is available. Irinotecan dose should be reduced by 30% at Cycle 1 treatment in poor metabolizers for all indications, with doses titrated thereafter based on tolerability and neutrophil counts. The lack of evidence precludes us from making any recommendation for rare malignancies such as sarcomas. Our guideline is consistent with other international pharmacogenetics prescribing guidelines. This guideline is grounded in the latest evidence but cannot account for all individual factors relevant to patient care. Therefore, prescribers must conduct a thorough assessment of each patient's risk–benefit profile, ensuring that therapy is optimized to maximize benefits while minimizing potential harms.

## 1 BACKGROUND AND OVERVIEW

Irinotecan, a topoisomerase I inhibitor, in its non‐pegylated formulation is licensed for the treatment of advanced colorectal cancer. Over time, however, its use has expanded, and it is now also used off‐label for pancreatic and upper gastrointestinal malignancies. Irinotecan can be prescribed as monotherapy or in combination with 5‐fluorouracil (5‐FU), for example, FOLFIRI, capecitabine, for example, CAPIRI, or with oxaliplatin, for example, FOLFOXIRI or FOLFIRINOX regimens.^1^, ^2^ Irinotecan has also been used off‐label in combination with other agents for the treatment of sarcomas, for example, with temozolomide for recurrent Ewing sarcoma.^3^ In its pegylated formulation, irinotecan is licensed for the treatment of advanced pancreatic adenocarcinoma in combination with other agents. At the time of writing, pegylated liposomal irinotecan has not been recommended for use in the National Health Service (NHS) by the National Institute for Health and Care Excellence (NICE).^4^ It is also important to note that the formulations are not interchangeable.

Irinotecan is a prodrug that is hydrolysed by hepatic and intestinal carboxylesterases to an active metabolite, SN‐38, which exhibits 100‐ to 1000‐fold greater cytotoxic potency.^5^, ^6^, ^7^ SN‐38 is inactivated by hepatic and extrahepatic uridine diphosphate glucuronosyltransferase (UGT) enzymes, mainly the UGT1A1 isoform.^8^ SN‐38 glucuronide (SN‐38G) then undergoes biliary excretion with potentially significant enterohepatic recirculation.^9^

The adverse effect profile of irinotecan includes an acute cholinergic syndrome as well as typical antiproliferative toxicities such as diarrhoea, alopecia, stomatitis and myelosuppression.^10^ The dose‐limiting toxicities of diarrhoea and neutropenia, can be graded between 1 and 5, using the Common Terminology Criteria for Adverse Events (CTCAE), with Grade 3 or 4 being reported in 21%–31% and 19%–31% of patients, respectively, when treated with weekly single‐agent irinotecan (85–125 mg/m^2^).^10^, ^11^ Severe toxicity may occur in the absence of clinically known risk factors, for example, hepatic and/or renal impairment, due to loss‐of‐function genetic variants within the *UGT1A1* gene, increasing SN‐38 exposure.^12^, ^13^, ^14^

*UGT1A1* loss‐of‐function alleles have been clinically associated with Gilbert's syndrome and Crigler‐Najjar syndrome.^15^ As described in Table 1, there are several allelic variants in the *UGT1A1* gene, occurring in either the promoter or coding regions (mostly exon 1 and 5) of the gene. UGT1A1*6, UGT1A1*28 and UGT1A1*37 are responsible for most cases of reduced UGT1A1 function and are the primary focus of current pharmacogenetic guidance.^14^, ^16^ Other loss‐of‐function variants (e.g., *7, *27) have been described, but their rarity limits the available clinical evidence.^14^ Gain of function variants such as UGT1A1*36 have also been described, but current evidence is insufficient to support their use in guiding irinotecan dose escalation, with the Dutch Pharmacogenetics Working Group (DPWG) recommending dosing as per the wild‐type UGT1A1*1 allele.^14^ The commonly studied loss of function allele is UGT1A1*28, with a minor allele frequency of 32%–36% in Europeans, 37% in African Americans/Afro‐Caribbean, 15% in East Asians and 41% in South Asians (Table 2).^17^, ^18^ Conversely, UGT1A1*6 is prevalent amongst Asian populations (2%–16%) but rarely found within Caucasian and African populations (0%–4%).^17^ Within Afro‐Caribbean/Afro‐American and African ancestries, the *37 allele is prevalent in 6% and 4%, respectively.^17^

**TABLE 1:** Common *UGT1A1* variants associated with irinotecan toxicity.

Gene	dbSNP	Chromosome change	Gene change	Alternative nomenclature	Amino acid change	Impact on enzyme	Clinical impact
UGT1A1*1	rs3064744	NC_000002.12:g.233760235TA[7]	NG_002601.2:g.175492TA[7]	(TA)_6_TAA A (TA)_6_TAA		Normal function	
TATA box polymorphism (TA repeat changes)							
UGT1A1*36	rs3064744	NC_000002.12:g.233760235TA[6]	NG_002601.2:g.175492TA[6]	(TA)_5_TAA A (TA)_5_TAA	Not applicable: variant occurs in a non‐coding region	Increased function	
UGT1A1*28	rs3064744	NC_000002.12:g.233760235TA[8]	NG_002601.2:g.175492TA[8]	(TA)_7_TAA A (TA)_7_TAA	Not applicable: variant occurs in a non‐coding region	Decreased function	Gilbert's syndrome
UGT1A1*37	rs3064744	NC_000002.12:g.233760235TA[9]	NG_002601.2:g.175492TA[9]	(TA)_8_TAA A (TA)_8_TAA	Not applicable: variant occurs in a non‐coding region	Decreased function	Neonatal hyperbilirubinemia
Polymorphisms within coding region (exon 1)							
UGT1A1*6	rs4148323	NC_000002.12:g.233760498G>A	NM_000463.3:c.211G>A		NP_000454.1:p.Gly71Arg	Decreased function	Gilbert's syndrome Neonatal hyperbilirubinemia

*Note*: Variants information obtained from Laval University, Quebec, Canada,^24^ and the ClinPGx database.^25^

**TABLE 2:** Population frequencies of the *UGT1A1* alleles.

Population	Allele frequency (%)			
	*6	*28	*36	*37
European (without Finland)	0.1	32		0.1
European (Finnish)	4.4	36	0	0
African American/Afro‐Caribbean	0.4	37	8	6
African	0	40	7	4
Near/Middle Eastern	0.2	31	1	1
East Asian	16	15	0	0
South Asian	2	41	0	0
Ashkenazi Jewish	0.4	38		

*Note*: Data obtained from gnomAD version 4.1 and ClinPGx *UGT1A1* allele frequency table.^17^, ^18^, ^26^

Promoter region variants impact a region containing thymine‐adenine repeats, also known as tandem repeats. The wild‐type allele (UGT1A1*1) has 6 thymine‐adenine (TA) repeats, with increased function being associated with fewer repeats, for example, UGT1A1*36 has 5 TA repeats (Table 1). Conversely, loss of function alleles is associated with an increased number of repeats, for example, UGT1A1*28 has 7 TA repeats within the TATA box resulting in a 35% reduction in gene transcription in heterozygotes and a 70% decrease in homozygotes.^19^, ^20^, ^21^ The lower gene expression seen in subjects with a UGT1A1*28/*28 genotype is associated with an 18%–159% increase in SN‐38 exposure and a reduction in metabolic clearance by 61%.^14^ Homozygous or heterozygous subjects for the UGT1A1*28 variant have an increased risk of developing neutropenia irrespective of the dose administered.^22^ In 2005, the Food and Drug Administration (FDA) issued the first pharmacogenomic recommendation that irinotecan dose reduction should be considered in individuals known to be UGT1A1*28 homozygotes.^23^

## 2 LICENSED INDICATIONS

Irinotecan is licensed in the United Kingdom for intravenous infusion as conventional non‐pegylated (CAMPTO and generics) and liposomal (ONIVYDE pegylated liposomal) formulations.

**Table:** 

Currently licensed indications for irinotecan in the UK^a^
Irinotecan (non‐pegylated) formulations^27^, ^a^
Irinotecan is indicated for the treatment of patients with advanced colorectal cancer: In combination with 5‐fluorouracil and folinic acid in patients without prior chemotherapy for advanced disease, As a single agent in patients who have failed an established 5‐fluorouracil containing treatment regimen. Irinotecan in combination with cetuximab is indicated for the treatment of patients with epidermal growth factor receptor (EGFR)‐expressing RAS wild‐type metastatic colorectal cancer, who had not received prior treatment for metastatic disease or after failure of irinotecan‐including cytotoxic therapy. Irinotecan in combination with 5‐fluorouracil, folinic acid and bevacizumab is indicated for first‐line treatment of patients with metastatic carcinoma of the colon or rectum. Irinotecan in combination with capecitabine with or without bevacizumab is indicated for first‐line treatment of patients with metastatic colorectal carcinoma.
ONIVYDE pegylated liposomal irinotecan^28^, ^a^
In combination with oxaliplatin, 5‐fluorouracil (5‐FU) and leucovorin (LV) for the first‐line treatment of adult patients with metastatic adenocarcinoma of the pancreas. In combination with 5‐FU and LV for the treatment of metastatic adenocarcinoma in adult patients who have progressed following gemcitabine‐based therapy.

^a^ The exact wording has been extracted from the Summary of Product Characteristics (SmPC) of the branded formulation of irinotecan CAMPTO (text revised April 2024) and pegylated liposomal formulation ONIVYDE (text revised March 2025).^27^, ^28^

### 2.1 Expansion of irinotecan indications

For the rest of this article, any reference to irinotecan relates to the non‐pegylated form, unless otherwise stated. As indicated above, irinotecan is used off‐label for a number of other malignancies, ranging from neoadjuvant to advance disease clinical settings (Table 3). Irinotecan can be prescribed in different ways, ranging from daily dosing to two‐ to four‐weekly regimens, and with varying doses, dependent upon the clinical indication, with higher doses being prescribed as three‐ to four‐weekly regimens. Table S1 provides a few examples of treatment schedules routinely prescribed within the United Kingdom.

**TABLE 3:** Clinical areas which consider the use of irinotecan.

Clinical system	Cancer
Gastrointestinal	Ampullary
	Cholangiocarcinoma
	Colorectal
	Gastric
	Oesophageal
	Pancreatic
	Small Bowel
	Neuroendocrine
Bone and soft tissue	Ewing sarcoma
	Rhabdomyosarcoma
Others	Cancer of unknown primary
	Glioblastoma
	Neuroblastoma
	Embryonal brain tumours, particularly medulloblastoma

## 3 EVIDENCE OVERVIEW

Over the past two decades, our understanding of the role of *UGT1A1* variants in determining interindividual variability in irinotecan response has progressed. Observational and pharmacokinetic studies have led to real‐world implementation studies examining the impact of genotype‐guided dose adjustments.

The 2023 DPWG guideline provides the most comprehensive literature review to date, covering 48 publications before March 2021.^14^ The authors reviewed diverse pharmacokinetic and observational research as well as 12 meta‐analyses assimilating data from thousands of patients, mostly from Caucasian and Asian populations.^14^

Meta‐analyses investigating the relationship of UGT1A1*28 and *6 alleles with irinotecan‐related adverse events have consistently found higher rates of serious toxicity in individuals carrying these alleles compared with those who are wild‐type (UGT1A1*1/*1).^22^, ^29^, ^30^, ^31^, ^32^, ^33^, ^34^, ^35^, ^36^ This applies to both heterozygote (intermediate metabolizers) and homozygote (poor metabolizers) carriers albeit to differing extents. UGT1A1 *28 and *6 alleles have similar functional effects on UGT1A1 activity, reflected by comparable SN‐38G/SN‐38 ratios.^37^ Conventional irinotecan dosing has historically been guided by the high prevalence (46%–55%) of *UGT1A1* *1/*28 intermediate metabolizers in Caucasian populations.^14^, ^38^ All major guidelines recognize a drug‐gene interaction for intermediate metabolizers, but there are no recommendations for dose reduction in this group due to concerns of adversely impacting efficacy. Dose reduction is thus reserved for poor metabolizers only.^38^ Conversely, the possibility of safely escalating irinotecan dosing in wild‐type patients has been suggested,^14^, ^38^ but not yet established. For instance, in a 100‐patient study that used first‐line FOLFIRI and bevacizumab for metastatic colorectal cancer, three dosing schedules for two‐weekly irinotecan were utilized: UGT1A1*1/*1 homozygotes were prescribed 310 mg/m^2^, UGT1A1*1/*28 heterozygotes were given 260 mg/m^2^, while a dose of 180 mg/m^2^ was administered to individuals with UGT1A1*28/*28 genotype, with the aim of prolonging PFS by increasing the dose.^39^ In the UGT1A1 *1/*1 and *1/*28 patients, the PFS was 12.5 and 14.6 months, respectively, indicating only a modest improvement in PFS at the expense of higher rates of Grade 3–4 neutropenia.^39^

Several meta‐analyses have found significantly higher rates of serious toxicity amongst UGT1A1 poor metabolizers.^22^, ^29^, ^30^, ^31^, ^32^ Most of these meta‐analyses have reported that UGT1A1 *28 and *6 variants were more strongly linked to CTCAE Grade 3 or 4 neutropenia than to diarrhoea and that the risk increases with higher irinotecan doses.^22^, ^29^, ^30^, ^31^, ^32^ In addition, two meta‐analyses found no increased risk of severe (CTCAE Grade ≥3) diarrhoea at low doses (defined as <150 or <125 mg/m^2^) while two did report an increased risk of severe neutropenia at these doses.^22^, ^30^, ^31^, ^32^

Pharmacokinetic studies support these findings and help guide genotype‐based dose adjustments. Data from six pharmacokinetic studies, including a total of 28 patients with the UGT1A1*28/*28 genotype, indicate that a mean dose adjustment of 69% of the standard irinotecan dose would be required to achieve the same SN‐38 exposure in these patients in comparison to the rest of the population (heterozygotes and wildtype) and a 58% reduction when compared with just wild‐type individuals. This underpins the DPWG recommendation to start such patients at 70% of the standard dose.^14^ Data published since the 2023 DPWG guideline have further confirmed these genotype–phenotype relationships by replicating the findings in Asian populations.^40^, ^41^, ^42^

A meta‐analysis of 42 Asian cancer studies found UGT1A1*6 or *28 homozygotes had a 6.23‐fold (*p* < .00001) increased risk of developing neutropenia and 3.21‐fold (*p* < .00001) increased risk of developing diarrhoea compared with wild‐type patients.^40^ A more recent meta‐analysis of 4471 colorectal cancer patients across 30 cancer centres, came to a similar conclusion: the odds ratio (OR) of *UGT1A1* *28 homozygotes developing neutropenia and diarrhoea was 4.10 (*p* < .0001) and 3.24, respectively (*p* < .0001).^42^ Similarly, the risk of developing diarrhoea (OR = 2.42; 95% confidence interval [CI] = 1.14–5.11, *p* = .03) and neutropenia (OR = 1.91; 95% CI = 1.17–3.10, *p* = .01, for neutropenia) was significantly increased in UGT1A1*6 homozygotes.^42^

### 3.1 Evidence for genotype‐guided irinotecan dosing

Latest research provides real‐world evidence regarding the safety, feasibility, pharmacokinetics, cost‐effectiveness and clinical efficacy of genotype‐guided irinotecan dose adjustment, although some uncertainty remains regarding optimal dosing.^43^, ^44^, ^45^, ^46^, ^47^, ^48^

A Dutch prospective multicentre clinical implementation study genotyped 350 adults receiving high‐dose (≥180 mg/m^2^ or 450–600 mg flat dose) irinotecan therapy. Homozygous subjects for the UGT1A1*28 and/or *93 allelic variants were defined as UGT1A1 poor metabolizers.^48^ Further dose optimisation was undertaken during subsequent cycles based on drug tolerability and neutrophil counts. UGT1A1*93 is in high linkage disequilibrium (LD) with UGT1A1*28 (*r* ^2^ = .83) and associated with an increased risk of irinotecan haematological toxicity.^49^ This Dutch cohort primarily consisted of Caucasian patients receiving FOLFIRI or FOLFIRINOX regimens for colorectal or pancreatic cancer. Poor metabolizers (*n* = 31) were compared against historical poor metabolizer controls. Febrile neutropenia was reduced from 24% to 7% (*p* = .04) in poor metabolizers treated with a reduced dose in comparison with historical poor metabolizer controls.^48^ This risk of febrile neutropenia was similar to non‐poor metabolizers treated at standard doses (7% *vs*. 4%) and *UGT1A1* poor metabolizers still had higher CTCAE Grade ≥3 toxicities overall (58% *vs*. 35%, *p* = .01).^48^ Concomitant pharmacokinetic data showed that systemic exposure to SN‐38 remained 32% higher in this patient subgroup than the standard dosed cohort (*p* = .05), thus explaining the higher rate of toxicity reported.^48^

Personeni et al. examined the rates of toxicity over 8 years across 247 Italian patients with gastrointestinal malignancies.^44^ The authors identified that of the poor metabolizers (UGT1A1*28/*28; *n* = 28*)* who received a median 40% (22%–58%) irinotecan dose reduction, 39.2% developed CTCAE Grade ≥3 neutropenia compared with only 19.6% of heterozygous or wild‐type patients treated at full dose (*p* = .058, *n* = 219).^44^

In a study that recruited 620 *UGT1A1*‐genotyped patients within the phase III AXEPT trial across 98 hospitals in Japan, China and South Korea, poor metabolizers were more likely to develop CTCAE Grade ≥3 neutropenia in the early treatment cycles, especially within the FOLFIRI cohort.^45^ The study compared XELIRI (CAPIRI) (irinotecan 200 mg/m^2^ every 3 weeks) with FOLFIRI (irinotecan 180 mg/m^2^ every 2 weeks) as second‐line therapy for metastatic colorectal cancer.^45^ Within the study, poor metabolizers (n = 47) received a reduced irinotecan dose of 150 mg/m^2^ in both arms equating to a 25% and 16.6% cumulative dose reduction for the XELIRI (CAPIRI) and FOLFIRI regimens, respectively.^45^ In a Japanese study, Satake et al. retrospectively analysed the clinical outcomes of 235 *UGT1A1*‐genotyped patients with advanced pancreatic cancer receiving FOLFIRINOX.^46^ UGT1A1 poor metabolizers (*6/*6 and *28/*28, *n* = 11) started with a lower irinotecan dose, reduced from 150 to 80 mg/m^*2*^ every 2 weeks.^46^ Amongst the 11 poor metabolizer patients, two patients developed CTCAE Grade ≥3 neutropenia and one patient had Grade 3 febrile neutropenia, but the overall rates of Grade 3–4 neutropenia/febrile neutropenia were lower in comparison to non‐poor metabolizers when the lower 80 mg/m^*2*^ dose was applied.^46^

There are limited data on genotype‐guided‐dosing for glioblastoma and for the lower dose protracted schedules typically used in paediatric practice and in the treatment of ‘paediatric’ cancers such as Ewing sarcoma in adults. Multiple studies have examined protracted schedules in medulloblastoma, rhabdomyosarcoma, neuroblastoma and Ewing sarcoma.^50^, ^51^, ^52^, ^53^ In these settings, irinotecan is used at lower doses and often administered orally rather than intravenously. For example, in recurrent/progressive Ewing sarcoma, irinotecan was administered as 10 to 20 mg/m^2^ in a 5‐day or 10‐day schedule (in combination with temozolomide), every 3 weeks. For relapsed rhabdomyosarcoma 50 mg/m^2^ on Days 1 to 5 is commonly prescribed (in combination with temozolomide and vincristine), every 3 weeks, with weekly cumulative doses ranging between 50 and 250 mg/m^2.^ ^52^, ^54^ Within paediatric irinotecan protocols, patients are given an oral cephalosporin (usually starting 2 days before chemotherapy and continued until 3 days after finishing chemotherapy). This has been reported to reduce the incidence of CTCAE Grade 3–4 irinotecan‐induced diarrhoea by reducing production of SN‐38 locally in the gut by bacterial glucuronidases.^55^ Paediatric studies of irinotecan have reported that the main toxicity is diarrhoea, usually managed as an outpatient, with myelosuppression being uncommon.^55^ Furthermore, an evaluation of 74 paediatric patients who received irinotecan (15–75 mg/m^2^) showed that severe toxicity was not increased in UGT1A1*28 homozygotes, despite a higher area under the curve for SN‐38.^56^ The major limitation in this area is the small sample sizes, which have been studied together with confounding caused by variable (and lower) dosing regimens, oral administration of irinotecan, concomitant use of a cephalosporin, different concomitant chemotherapeutic drugs and limited assessment of the correlation between genotype and irinotecan pharmacokinetics.

For glioblastomas, irinotecan can also be prescribed with variable dosing schedules ranging from 125 mg/m^2^ every 2 weeks to 350 mg/m^2^ every 3 weeks.^57^ Very few studies have evaluated the effect of *UGT1A1* genotype in patients receiving irinotecan for the treatment of glioblastoma. Interestingly the role of *UGT1A1* genotype may also be modulated by using enzyme‐inducing anti‐seizure medications. For instance, one study showed that UGT1A1*28 status was important in determining toxicity in patients not receiving enzyme inducing anti‐seizure medications when compared with patients concomitantly receiving the enzyme inducer despite having been given an approximately three‐fold lower irinotecan dose.^58^ A Phase 1 study of intravenous liposomal irinotecan excluded UGT1A1*28 homozygous patients and compared heterozygotes given 60 mg/m^2^ irinotecan every 3 weeks (with dose increases in 30‐mg/m^2^ increments) and wild‐type homozygotes given 120‐mg/m^2^ irinotecan every 3 weeks (with dose increases in 60‐mg/m^2^ increments). The two groups had equivalent pharmacokinetic profiles and there was no correlation with toxicity.^59^

### 3.2 *UGT1A1* genotyping and clinical efficacy

Early studies suggested that higher SN‐38 levels, the active irinotecan metabolite, due to genetically reduced UGT1A1 activity, might enhance tumour response,^60^, ^61^ but subsequent studies have shown that this does not necessarily translate to improved outcomes such as survival.^33^, ^62^, ^63^, ^64^ A recent meta‐analysis that included 282/912 UGT1A1*6 and 281/1025 UGT1A1*28 carriers from across 11 and 12 Chinese studies, respectively, confirmed no benefit in remission rates in UGT1A1 intermediate/poor metabolizers compared with wild‐type patients.^41^ In clinical practice, severe toxicity associated with reduced UGT1A1 activity may results in dose de‐escalation, delay or cessation of further chemotherapy cycles compromising effective treatment.

Randomized control trials directly comparing genotype‐guided against standard irinotecan dosing are lacking, as withholding dose reduction in UGT1A1 poor metabolizers would be unethical. Three recent studies (Table 4) assessed the impact of genotype‐guided dose adjustments through comparing survival outcomes by genotype.^45^, ^46^, ^47^ Lv et al. treated 107 Chinese gastric cancer patients with genotype‐based dosing with no differences in median overall survival (OS) between wild‐type (9.5 months) and intermediate metabolizers (9.0 months) compared with poor metabolizers (11.5 months, *p* = .528).^47^ Similar results were also reported by Satake et al. in a cohort of 235 patients with a reported median OS of 8.8 months in non‐poor metabolizers compared with 11.5 months in poor metabolizers (*p* = .579).^46^ Conversely, the AXEPT III clinical study showed that median OS was reduced (*p* = .008) in dose‐reduced poor metabolizers with the same trend for PFS and objective response rate (ORR).^45^ The authors explained that this difference may be related to the irinotecan dose still being too high for poor metabolizers at 150 mg/m^2^ within the FOLFIRI arm, leading to the higher discontinuation rates due to early development of Grade 3–4 neutropenia.^45^

**TABLE 4:** Survival outcomes in cohorts who have received *UGT1A1* genotype‐guided dose reductions.

Study	Cancer type	*N* ^a^	Irinotecan dose			Median overall survival (months)			*p* value^b^
			WT	IM	PM	WT	IM	PM	
Iwasa et al. (2021)^43^	Colorectal	650	200 mg/m^2^ (CAPIRI)^c^ every 3 weeks OR 180 mg/m^2^ (FOLFIRI)^c^ every 2 weeks		150 mg/m^2^	15.9	17.7	10.6	.008
Satake et al. (2022)^44^	Pancreatic	235	150 mg/m^2^ (FOLFORINOX)^c^ every 2 weeks		80 mg/m^2^	8.8		11.5	.579
Lv et al. (2024)^45^	Gastric	107	125 mg/m^2^	100 mg/m^2^	75 mg/m^2^	9.5	9.0	11.2	.528
Peeters et al. (2026)^f^ ^,^ ^65^	Colorectal or pancreatic	779	180 mg/m^2^ (FOLFIRINOX)^c^ every 2 weeks; 150 mg/m^2^ (modified FOLFIRINOX)^c^ every 2 weeks; 180 mg/m^2^ or 450 mg flat dose (Low dose irinotecan monotherapy)^d^; 350 mg/m^2^ or 600 mg flat dose (high dose irinotecan monotherapy)^e^; 180 mg/m^2^ (FOLFIRI)^c^ every 2 weeks; 180 mg/m^2^ (FOLFIRI)^c^ + bevacizumab/cetuximab every 2 weeks; 165 mg/m^2^ (FOLFOXIRI)^c^ every 2 weeks; 165 mg/m^2^ (FOLFOXIRI^c^ + bevacizumab) every 2 weeks; other^c^		30% dose reduction applied	Colorectal 14.9; pancreatic 14.8		Colorectal 13.4; pancreatic 11.6	.42

^a^ *N* = number of patients; WT = wild‐type; IM = intermediate metabolizers; PM = poor metabolizers; WT (normal metabolizers) = UGT1A1*1/*1; IM = UGT1A1*1/*6, *1/*28; PM = UGT1A1*6/*6, *28/*28,*6/*28.

^b^ *p* value compares median overall survival between poor metabolizers (PMs) against both wild‐type and intermediate metabolizers (IMs).

^**c**^ XELIRI (also known as CAPIRI within the NHS) = irinotecan 200 mg/m^2^ (poor metabolizers within AXEPT III study received 150 mg/m^2^), capecitabine 800 mg/m^2^ twice daily on days 1–14 every 3 weeks; FOLFIRI = irinotecan180 mg/m^2^ (poor metabolizers within AXEPT III study received 150 mg/m^2^), 5‐fluorouracil 400 mg/m^2^, 5‐fluorouracil 2400 mg/m^2^ every 2 weeks; FOLFIRINOX = irinotecan 150 mg/m^2^, oxaliplatin 85 mg/m^2^, 5‐fluorouracil 400 mg/m^2^ and 5‐fluorouracil 2400 mg/m^2^ every 2 weeks.

^d^ Irinotecan monotherapy low dose: 180 mg/m^2^ or 450 mg flat dose either every 2 or 3 weeks.

^e^ Irinotecan monotherapy high dose: 350 mg/m^2^ or 600 mg flat dose either every 2 or 3 weeks.

^f^ Within Peeters et al., other listed as irinotecan monotherapy 210 mg/m^2^, irinotecan + panitumumab 180 mg/m^2^ every 2 weeks, FOLFIRI + panitumumab 180 mg/m^2^ every 2 weeks; FOLFIRI 180 mg/m^2^ every 3 weeks, FOLFIRI + ramucirumab 180 mg/m^2^ every 2 weeks, mFOLFOXIRI + panitumumab 150 mg/m^2^ every 2 weeks, irinotecan + cetuximab 150 mg/m^2^ every 2 weeks, irinotecan + bevacizumab 180 mg/m^2^, irinotecan monotherapy 240 mg flat every 2 weeks.^65^

More recently, a large retrospective multicentre cohort study (*n* = 779) reported no significant differences in PFS or OS between poor metabolizers (*n* = 76) receiving an initial 30% dose reduction and intermediate or normal metabolizers (*n* = 703) prescribed full dose over a median follow‐up of 23.4 months.^65^ The poor metabolizer cohort demonstrated a median PFS of 6.2 months (OS: 13.4 months) in colorectal cancer and 9.0 months (OS: 11.6 months) in pancreatic cancer.^65^ In comparison, over a median follow‐up of 28.8 months, the intermediate and normal metabolizers showed a median PFS of 6.0 months (median OS: 14.9 months) for colorectal and 8.3 months (median OS: 14.8 months) for pancreatic cancer (adjusted hazard ratio 1.08 [95% CI: 0.84–1.40; *p* = .54] for PFS and 1.13 [95% CI: 0.84–1.51; *p* = .42] for OS).^65^ Notably, 92% of the study population was of European ancestry, which may limit the generalizability of these findings to populations where variants such as UGT1A1*6 are more prevalent.

### 3.3 Clinical feasibility data

Four studies (Table 5) reported the clinical feasibility of *UGT1A1* genotyping in terms of speed of testing, which is highly variable depending on various logistical factors including location of testing.^43^, ^48^, ^66^, ^67^ A prospective randomized implementation study assessed the feasibility of pre‐emptive *DPYD* and *UGT1A1* testing in 288 patients in Philadelphia, USA.^43^ The authors reported 57% of results were available when commencing treatment, with a median turnaround time of 10 days.^43^ A retrospective review of 280 genotyped patients from a community‐based cancer centre in Cincinnati, USA, found the median turnaround time was much shorter at seven calendar days.^66^

**TABLE 5:** Studies reporting clinical feasibility of *UGT1A1* genotyping.

Study	Location	Number of patients genotyped	Median turnaround time	Results received before commencing treatment (%)
Hulshof et al. (2022)^48^	The Netherlands	350	Not reported	99.7
Muldoon et al. (2024)^a^ ^,^ ^66^	Cincinnati, USA	280	7	77.9
Glewis et al. (2024)^a^ ^,^ ^67^	Victoria, Australia	50	7	96.0
Tuteja et al. (2025)^a^ ^,^ ^43^	Philadelphia, USA	288	10	57.4

^a^ Tuteja et al. (2025), Muldoon et al. (2024) and Glewis et al. (2024) reported both *DPYD* and *UGT1A1* genotyping together.^43^, ^66^, ^67^

### 3.4 Summary of evidence

The relationship between UGT1A1 *6 and *28 homozygosity and increased irinotecan toxicity is established in the treatment of epithelial cancers. A multitude of meta‐analyses have demonstrated this relationship for a wide range of solid tumours across both Caucasian and Asian populations. Genotype‐guided irinotecan dose reduction for *UGT1A1* PM reduces the rates of toxicity, although some uncertainty remains regarding optimal dosing.^44^, ^45^, ^46^, ^48^ Despite upfront dose reductions ranging from 16% to 40%, an excess of toxicity was still observed amongst poor metabolizers, with only one study which almost halved irinotecan dose reported toxicities returning to baseline.^46^ Overall, these findings suggest that the 30% reduction recommended by the DPWG may be insufficient to fully mitigate toxicity risk but is a reasonable starting point to initiate a dose reduction.^14^ Data on clinical outcomes by *UGT1A1* genotype suggest that toxicity and efficacy are not in direct opposition and may be reconciled.^41^ However, more prospective evidence on outcomes for PFS and OS, across more diverse patient populations is needed to determine the optimal dose for each genotype. Real‐world data have demonstrated clinical feasibility with rapid turnaround times and the cost‐saving effects of implementation.^43^, ^48^, ^66^ For non‐epithelial cancers, variable and usually lower weekly irinotecan dosing schedules are prescribed, for example, 20 to 100 mg/m^2^; therefore, further data are required to assess the utility of *UGT1A1* genotyping.

## 4 RECOMMENDED INDICATIONS FOR PHARMACOGENETIC TESTING

### 4.1 Epithelial cancers

*UGT1A1* testing should be offered to all patients who are due to receive irinotecan‐based regimens, irrespective of their ancestry.

### 4.2 Non‐epithelial cancers

There is inadequate evidence to recommend the routine pre‐prescription use of *UGT1A1* genotyping in patients receiving irinotecan for non‐epithelial malignancies, where per‐day doses are usually lower than in epithelial cancers. Where higher doses of irinotecan are used, for example, for the treatment of glioblastoma multiforme, the use of genotyping is optional and further evidence is required in this patient group.

### 4.3 History of severe toxicity

Genotyping should be considered in patients of any age with any malignancy where severe toxicity (Grade 3 or higher) has been seen with the use of irinotecan, irrespective of dose.

Pharmacogenetic testing may not be required if genetic information is already available in the patient's medical records. The availability of genetic testing may vary in time, indication and in geography. In the absence of available relevant pharmacogenetic information or testing, the current best practice clinical guidelines should be followed.

## 5 INTEGRATING PHARMACOGENETIC TESTING INTO EXISTING CLINICAL PATHWAYS

*UGT1A1* pharmacogenetic testing should be requested prior to the treatment of an irinotecan‐based regimen for epithelial cancers. Those who are identified as poor metabolizers should be offered a dose reduction at Cycle 1 where clinically appropriate, with the dose titrated based on tolerability and neutrophils counts.

The results should be placed and available where all healthcare professionals involved in the prescribing, validation and administration of irinotecan can view the results. It is recommended that the results should be placed alongside other pharmacogenetic results, for example, *DPYD*. Results recorded by the receiving clinician should ideally be in the form of structured data where these are available, for example, using SNOMED‐CT codes (Table 6). Therefore, laboratories may wish to highlight these codes within results.

**TABLE 6:** SNOMED‐CT codes.

SNOMED‐CT code wording	SNOMED CT code ID
Uridine diphosphate glucuronosyltransferase family 1 member A1 **normal metabolizer** (finding)	738 538 001
Uridine diphosphate glucuronosyltransferase family 1 member A1 **intermediate metabolizer** (finding)	738 537 006
Uridine diphosphate glucuronosyltransferase family 1 member A1 **poor metabolizer** (finding)	738 536 002

It is also best practice to provide patients with information regarding the purpose of the test (refer to Supporting information S1 Plain English Summary), the rationale for dose modifications based on the pharmacogenetic test results and the local turnaround time to obtain those results.

## 6 WHICH GENES, VARIANTS, TURNAROUND TIME?

The *UGT1A1* variants to be tested include *6, *28, *36 and *37. As described in Table 1, the variants *28, *36 and *37 are found within the TATA section of the 5′ promoter region while the exonic *6 variant is a missense variant because of the amino acid change. The full molecular descriptions of the *UGT1A1* alleles have been described by the Laval University, Quebec, Canada.^24^ The allelic frequencies are described (Table 2), with the most common genotype/phenotype examples provided within Table 7. It is important to note that implementation requirements will need to be updated with relevant changes in genotype‐to‐phenotype translations and allele definitions. This information can be obtained by the allele function assignments provided by Clinical Pharmacogenetics Implementation Consortium (CPIC) in the diplotype–phenotype tables available on the ClinPGx database.^18^ Additional information on other *UGT1A1* allelic variants can be found in the ‘*UGT1A1* frequency table’ available through the ClinPGx database.^18^

**TABLE 7:** Frequencies of *UGT1A1* phenotype across different populations/geographical areas.

Genotype	Phenotype as per CPIC	Frequency of genotype/phenotype (%) in different populations^a^			
		Caucasian	African‐American	African^b^	Asian
*1/*1	Normal metabolizer	34–38	26	20–60	25–75
*1/*36 ^*e*^	Normal metabolizer	0–2	0–2	4–12	0
*1/*28	Intermediate metabolizer	46–55	33–37	30–50	15–60
*1/*37	Intermediate metabolizer	0	4–15	3–8	0
*1/*6	Intermediate metabolizer	1			4–28
*28/*36	Intermediate metabolizer	0	5	7–8	0
*36/*37	Intermediate metabolizer	0	3	1–2	0
*28/*28 ^c^	Poor metabolizer	11–13	13–19	6–18	2–20
*6/*6 ^d^	Poor metabolizer	0			0.3–4
*6/*28	Poor metabolizer				
*28/*37	Poor metabolizer	0–2	5–6	1–14	0

^**a**^ This table is adapted from the DPWG guideline data and separate genotype ancestry data for East or South Asian ancestry was not provided.^14^

^**b**^ African populations are not homogenous and thus UGT1A1*28/*28 frequencies differ across the African subcontinent, for example, reported as 8% within Egyptian and Côte d'Ivoire ancestries, 18% in Kenyans (Luo) and 6% in Madagascan populations.^68^

^**c**^ UGT1A1*28/*28 frequencies reported in the literature are 1%–2% for Chinese, <3% Japanese and 13% for Indian ancestries.^68^

^**d**^ UGT1A1*6/*6 frequencies are reported as 2% for Indians, 3% Chinese, 4% within Japanese and 3% observed within Malaysian populations.^69^

^e^ UGT1A1*36 is reported as a variant with increased function in the ClinPGx database. However, the diplotypes UGT1A1*1/*36 and UGT1A1*36/*36 are currently translated into normal metabolizer phenotypes.^18^

Pharmacogenetic testing for *UGT1A1* genetic variants should be undertaken by laboratory testing following similar procedures and pathways currently in place for *DPYD* testing. This will ensure that dosing decisions can be made prior to all Cycle 1 treatments. Therefore, laboratory service providers, irrespective of whether they are local, regional or national, should aim for a turnaround time of 5 days from receipt of the sample, which is the current best practice timeframe for *DPYD* testing. This timepoint is from the time the sample is taken, received and processed by the laboratory to the results being returned to the clinician for clinical action prior to treatment administration.^70^ It is recommended that clinicians should wait for the result before prescribing irinotecan. For treatments containing 5‐FU or its derivatives, clinicians should wait for both the *UGT1A1* and *DPYD* test results especially where the regimens contain both irinotecan and 5‐fluoropyrimidines, for example, gastrointestinal malignancies. Clinicians should consider that both tests are designed to identify a limited number of the most common allelic variants therefore reduced enzymatic activity due to rare variants may not be detected. This represents a known limitation of pharmacogenetic tests based on selected variants.

## 7 CLINICAL ACTIONS BASED ON GENOTYPE

Prescribing recommendations in epithelial cancers should be guided by the pharmacogenetic test results as per the Table 8.

**TABLE 8:** Recommended clinical actions in epithelial cancers based on the pharmacogenetic test results for *UGT1A1*.

Metabolizer phenotype	Prescribing suggestions for standard non‐pegylated irinotecan formulation
Normal metabolizer	No dose adjustments recommended at Cycle 1
Intermediate metabolizer	No dose adjustments recommended at Cycle 1
Poor metabolizer	Initiate 30% dose reduction at Cycle 1, with doses to be titrated based on tolerability and neutrophil counts.

In the event of delayed results in which the *UGT1A1* status is not yet known and to prevent delays to initiating treatment, an *optional* recommendation is that the first cycle should be initiated with a 30% dose reduction, with the dose then escalated to 100% for normal and intermediate metabolizers. The dose for poor metabolizers for subsequent cycles can be titrated as per patient tolerability and neutrophil counts. This is current practice in the Netherlands (van Schaik, personal communication) and is based on new evidence showing that a 30% dose reduction in poor metabolizers results in toxicity rates that are comparable with those observed in intermediate and normal metabolizers prescribed the full dose of irinotecan.^71^

For pegylated liposomal irinotecan (ONIVYDE) being used in combination with 5‐FU and leucovorin, the summary of product characteristics recommends ‘A reduced starting dose of ONIVYDE pegylated liposomal of 50 mg/m^2^ should be considered for patients known to be homozygous for the UGT1A1*28 allele. A dose increase of ONIVYDE pegylated liposomal to 70 mg/m^2^ should be considered if tolerated in subsequent cycles’.^28^

## 8 OTHER PHARMACOGENETICS GUIDELINES

### 8.1 The Genomic Applications in Practice and Prevention Working Group guideline

In 2009, the Genomic Applications in Practice and Prevention (EGAPP) Working Group published an evaluation of UGT1A1*28 genotyping in Caucasian patients^72^ and stated that the evidence was insufficient to recommend for or against the routine use of *UGT1A1* genotyping in patients with metastatic colorectal cancer being considered for treatment with irinotecan. Although there was convincing evidence of a significant association between UGT1A1*28 genotype and the incidence of severe neutropenia at standard irinotecan doses, the association with severe diarrhoea was not as strong. The guideline also concluded that there was lack of sufficient evidence (i.e., prospective studies, evidence on other alleles and impact on efficacy) for the clinical utility of the *UGT1A1* testing. This guideline has not been updated since 2009.

### 8.2 The French National Pharmacogenetics Network and the Group of Clinical Onco‐Pharmacology guidelines

The French National Pharmacogenetics Network (RNPGx) and the Group of Clinical Onco‐Pharmacology (GPCO‐Unicancer) first issued a guideline in 2015,^73^ based on a systematic literature review including studies evaluating (a) the association between *UGT1A1* variants and efficacy clinical outcomes in patients with metastatic or stage III colorectal cancer, (b) the relationship between *UGT1A1* variants and irinotecan toxicity mainly in patients with metastatic, advanced or Stage III colorectal cancer, (c) studies on genotype‐directed dose adjustments and (d) two meta‐analyses on irinotecan efficacy and three meta‐analyses on irinotecan toxicity in relation to the *UGT1A1* status.^22^, ^31^, ^32^, ^74^, ^75^ This guideline recommended pretreatment *UGT1A1* genotyping according to two levels of analyses. First‐line analyses included genotyping for the commonly identified TATA box variants (*28, *36 and *37) for all patients scheduled to receive irinotecan doses ≥180 mg/m^2^.^73^ Second‐line analysis focused on an extended panel of variants, which included the entire *UGT1A1* coding region, including *6 and *27 variants in patients of Asian origin. Genotyping was strongly advocated for patients scheduled for high‐dose irinotecan regimens (≥240 mg/m^2^, FOLFIRI‐HIGH), with the prescribing recommendations as follows:

- For doses >180 to 230 mg/m^2^, two‐ to three‐weekly (FOLFIRI), *UGT1A1* genotyping was recommended. Patients identified as*28/*28 to be prescribed a 25%–30% dose reduction at cycle 1, with the dose to be titrated based on tolerability.
- *UGT1A1* genotyping was strongly recommended for doses ≥240 mg/m^2^, two‐ to three‐weekly (FOLFIRI‐HIGH). Irinotecan at such an intensified dose is contraindicated in patients identified as UGT1A1*28/*28. However, those patients identified as *1/*28 should have a 30% dose reduction at Cycle 1, with the option to titrate based on tolerability.

The French guideline was updated in 2017 confirming previous recommendations.^76^ Therefore, *UGT1A1* genotyping to detect the *28 variant is advisable for a standard irinotecan dose (180–230 mg/m^2^) and essential for the intensified dose (>240 mg/m^2^).^77^ For the other *UGT1A1* alleles, genotyping is considered a second‐line test and ‘*it is performed by a limited number of laboratories in France*’. The literature review included two additional papers focusing on dose‐adjustments based on genotyping results.^78^, ^79^ Within the French guideline, dosing recommendation were predominantly in relation to colorectal cancer treatment regimens.

### 8.3 The Dutch Pharmacogenetics Working Group guidelines

The DPWG included prescribing recommendations based on the association between irinotecan and *UGT1A1* in the first pharmacogenetic prescribing guideline published in 2011.^80^ After a systematic literature review, the DPWG recommendation was to reduce irinotecan starting dose by 30% in UGT1A1*28 homozygous patients treated with the drug at doses higher than 250 mg/m^2^. The level of evidence was based on published controlled studies of moderate quality, but the clinical relevance was significant.^81^ The clinical relevance was scored on a seven‐point scale, adapted from the National Cancer Institute's Common Toxicity Criteria. A further update issued in 2018 confirmed that serious and life‐threatening adverse events occur more often in UGT1A1*28 homozygous patients. Evidence was generated mainly in the setting of metastatic or advanced colorectal cancer with some studies focusing on other epithelial cancers, including but not limited to gastric, gastrointestinal, oesophageal, lung cancer (small and non‐small cell lung cancer), gynaecological cancers and other solid tumours. Thus, the main recommendations did not vary from the 2011 guidance, that is, start with 70% of the normal dose in patients who are *UGT1A1* poor metabolizers (*28/*28 or *6/*6). At variance with the French guideline, the DPWG recommended dose adjustment of irinotecan in poor metabolizers for doses both above and below 180 mg/m^2^, based on the evidence from two meta‐analyses.^22^, ^31^ In addition, the DPWG has recently provided a clinical implication score, thus providing a direct evaluation of the clinical utility of pharmacogenetic testing. In this regard, *UGT1A1* genotyping for irinotecan is considered ‘essential’, meaning that *UGT1A1* pharmacogenetic testing must be performed prior to initiating irinotecan treatment.^14^, ^82^ It should be noted that sarcoma treatment regimens were not specifically mentioned within the guideline.

The DPWG has recently published an updated guideline.^14^ This updated version includes the *UGT1A1* alleles with minor allelic frequencies (MAF) ≥1% in either White, African and Asian populations and genotype to predicted phenotype translation to be programmed into laboratory information systems:

- UGT1A1*27 (rs35350960);
- UGT1A1*6 (rs4148323 and most prevalent in the Asian population);
- UGT1A1*28 or *37 (rs8175347).

These allelic variants were genotyped in the PREemptive Pharmacogenomic Testing for Preventing Adverse Drug REactions (PREPARE) study, which assessed the impact of pre‐emptive pharmacogenomic panel testing on the incidence of adverse drug reactions.^83^

### 8.4 The Italian Association of Medical Oncology and the Italian Society for Pharmacology guideline

In 2019, the Italian Association of Medical Oncology (AIOM) and the Italian Society for Pharmacology (SIF) published a *UGT1A1* guideline.^84^ This guideline is only available in Italian through the Societies' website, but excerpts from this guideline are accessible at ClinPGx.^85^ Pretreatment genotyping was recommended for all patients with clinical characteristics (comorbidity, performance status and disease stage) suggesting limited beneficial effects of irinotecan in terms of survival and/or response and/or at high risk/benefit ratio, according to the evaluation of the medical oncologist. The main prescribing recommendation was to use 70% of standard dose in UGT1A1*28 homozygous subjects (no other variants were mentioned). Genotyping was also recommended during therapy in any case of gastrointestinal toxicity (Grade ≥3) or haematological toxicity (Grade 4, NCI‐CTCAE v4.0) and in any case of serious unexpected toxicities.

### 8.5 The Clinical Pharmacogenetic Implementation Consortium

At present, the CPIC does not have a published guideline on *UGT1A1* and irinotecan but have labelled irinotecan and *UGT1A1* genotyping as a provisional level A test as the guidelines are currently within development. Based on this provisional level A grading, this means that genetic information should be used to change prescribing of the affected drug in the clinical context, as the current level of published evidence is considered moderate or strong in favour of changing prescribing.^18^

Table 9 provides a summary of current PGx guidelines.

**TABLE 9:** Summary of therapeutic recommendations for conventional irinotecan based on different guidelines.

UGT1A1 phenotype	EGAPP	GPCO‐UniCancer/RNPGx^a^	DPWG	AIOM/SIF	UK CERSI‐PGx^b^ (for epithelial cancers)
Normal metabolizer	No recommendation	>180 to 230 mg/m^2^ No action required ≥240 mg/m^2^ No action required	No action required	No action required	No dose adjustments recommended at Cycle 1
Intermediate metabolizer	No recommendation	>180 to 230 mg/m^2^ 30% dose reduction at Cycle 1 Dose titrated based on tolerability ≥240 mg/m^2^ 30% dose reduction at cycle 1	No action required	No action required	No dose adjustments recommended at Cycle 1
Poor metabolizer	No recommendation	>180 to 230 mg/m^2^ 25–30% dose reduction at Cycle 1 Dose titrated based on tolerability ≥240 mg/m^2^ Irinotecan contraindicated	30% dose reduction at Cycle 1 dose titrated based on tolerability	30% dose reduction at Cycle 1	Initiate 30% dose reduction at Cycle 1, with doses to be titrated based on tolerability and neutrophil counts.

^a^ All doses relate to two‐ to three‐weekly treatment schedules. *UGT1A1* genotyping is advisable for a standard dose (180–230 mg/m^2^) and essential for intensified dose (>240 mg/m^2^).

^b^ This dosing guideline is for the conventional non‐pegylated irinotecan formulation and clinical use of irinotecan in epithelial cancer. For ONIVYDE pegylated liposomal irinotecan the clinician will need to refer to the Summary of Product Characteristics.

## 9 HEALTH ECONOMIC EVALUATION

Six relevant cost–utility analyses were identified, two from the United Kingdom,^86^, ^87^ two from the United States^88^, ^89^ and one each from Germany^90^ and China.^91^ Five studies examined UGT1A1*28 ^86^, ^87^, ^88^, ^89^, ^90^ and one considered an additional variant, UGT1A1*6.^91^ Irinotecan was prescribed for metastatic colorectal cancer either as monotherapy^86^, ^88^ or in combination with other.^86^, ^87^, ^89^, ^90^, ^91^ Each study compared pharmacogenomic testing followed by a reduced irinotecan dose (20% to 50% of the standard dose) with standard care for patients who tests positive for *UGT1A1* variants. The standard irinotecan dose ranged from 175 to 350 mg/m^2^.

All studies used decision‐analytic models to assess cost‐effectiveness and adopted the perspectives of healthcare systems^86^, ^87^, ^91^ and payers.^88^, ^89^, ^90^ Both UK models adopted a lifetime horizon of analysis whereas others used shorter durations, ranging from 6 to 24 months^89^, ^91^; two studies did not specify the time horizon.^88^, ^90^

Amongst the UK studies, Hughes et al.^86^ evaluated the cost‐effectiveness of *UGT1A1* testing based on treatment pathways of the MRC FOCUS study^92^ and the risk of neutropenia and diarrhoea in patients with advanced colorectal cancer. Reactive testing, with a 20% dose reduction for patients who were homozygous or heterozygous for UGT1A1*28, was compared with standard care (no testing). Patients receiving standard care or with wild‐type alleles received standard doses of irinotecan—350 mg/m^2^ as monotherapy and 180 mg/m^2^ in combination. In the base‐case analysis, testing was dominated by standard care, being associated with higher mean costs (ranging from £54 to £63, depending on the treatment strategy) and marginally fewer lifetime quality‐adjusted life years (QALYs; less than one quality‐adjusted day). In a scenario where dose reduction was implemented only for patients homozygous for UGT1A1*28, testing was dominant in relation to second line irinotecan monotherapy (Modified de Gramont regimen), with a mean saving of £10 and 0.8 additional quality‐adjusted days (equivalent to £4323 saved for each QALY gained). When used at lower doses in combination with other chemotherapies (irinotecan modified de Gramont) regimen with or without first‐line doublet therapy, dose reductions in UGT1A1*28 homozygous patients were associated with increased costs (by £35) at reduced benefit (<0.001 QALYs).

Shabaruddin et al.^87^ evaluated the cost‐effectiveness of testing for *UGT1A1* in Caucasian patients with advanced colorectal cancer receiving second‐line irinotecan‐based chemotherapy based on the irinotecan modified de Gramont regimen. The economic model compared genotyping followed by a 20% dose reduction for patients homozygous for UGT1A1*28 with standard care (no genotyping and a standard irinotecan dose of 180 mg/m^2^). The model took into consideration the impacts of neutropenia on survival, costs and health utilities. *UGT1A1* testing was reported to be cost saving and to result in a lower incidence of severe neutropenia. For a cohort of 100 patients, the test was estimated to save £14 500, avoid 4.4 neutropenic episodes, gain 0.06 life‐years and 0.05 QALYs. A scenario analysis that considered febrile neutropoenia as the clinical outcome resulted in estimated savings (per 100 patients) of £17 706, avoidance of 0.38 Grade 3 or 4 neutropenic episodes and a gain of 0.007 QALYs.

Evaluations undertaken in other countries all found *UGT1A1* pharmacogenomic testing to be cost‐effective. Obradovic et al.^88^ reported cost‐effectiveness separately for Asian, African and Caucasian American populations, based on UGT1A1*28 homozygous genotype prevalences of 2%, 19% and 11%, respectively. Genotyping was both cost‐saving and more effective for populations of African (US $46 704 saved and 0.04 life‐years gained) and Caucasian ancestry (US $11 249 saved and 0.023 life‐years gained), but not amongst Asian populations (incremental cost‐effectiveness ratio of US $6 818 203 per life‐year gained, within more East‐Asian demographic) likely because of the lower prevalence of UGT1A1*28. Gold et al.^89^ evaluated the cost‐effectiveness of genotyping based on a 25% dose reduction (from 175 mg/m^2^) for UGT1A1*28 homozygous patients. Testing was found to be dominant, with a mean saving of US $272 and 0.0007 QALYs gained.

Compared with no testing, a strategy of a 25% dose reduction in UGT1A1*28 homozygous patients in Germany^90^ was dominant (saving €580 and 0.0002 QALY gained) and more cost‐effective than administering prophylactic granulocyte colony stimulating factors (GCSF) for homozygous and heterozygous patients. A decision analytic model by Wei et al.^91^ found that genotyping prior to irinotecan with dose adjustment (50% for UGT1A1*6/*28, UGT1A1*6/*6 or UGT1A1*28/*28) was both cost‐saving and more effective for Chinese patients. Compared with standard care, genotyping resulted in marginal QALYs increases (0.0011) and a mean cost reduction of $651 per patient.

## 10 REGULATORY CONSIDERATIONS

The UK CAMPTO SmPC for the conventional non‐pegylated irinotecan describes the role of *UGT1A1* variants increasing the risk of severe diarrhoea and neutropenia, especially at increased irinotecan dose levels, stated as >180 mg/m^2^ within section 4.4.^27^ Within section 5.2, reference is made to the well characterized variants UGT1A1*28 and UGT1A1*6 and it acknowledges the data associating an increased risk of neutropenia and diarrhoea in homozygote carriers, but no guidance is provided on recommended starting doses for poor metabolizers.^27^ In contrast, the SmPC (section 4.2) for ONIVYDE pegylated liposomal irinotecan provides clear dose reduction guidance for *UGT1A1* poor metabolizers stating a starting dose of 50 mg/m^2^, a 30% dose reduction from the licensed 70 mg/m^2^ dosing.^28^ This guidance is based on the NAPOLI‐1 randomized controlled trial where UGT1A1*28/*28 homozygote carriers were prescribed an upfront irinotecan dose reduction of 50 mg/m^2^.^28^ There is no other published UK‐based guidance outside of the SmPCs. With respect to other countries, within the United States, the FDA states that *UGT1A1* genotyping for *28 and *6 should be considered when irinotecan is being considered, with a one‐level reduction in the starting dose in poor metabolizers.^93^

## 11 OTHER CONSIDERATIONS

### 11.1 Gilbert's syndrome

Gilbert's syndrome is a common genetic condition caused by the *UGT1A1* variants *28 and *6. Genetic testing is available on the NHS for the purpose of diagnosing unconjugated hyperbilirubinemia in the absence of haemolysis and each nation will have eligibility criteria, which will need to be followed. *UGT1A1*, when used as a pharmacogenetic test, may identify patients with Gilbert's syndrome, and clinicians should follow NICE guidance and/or local practice guidance. Subjects with Gilbert's syndrome may be at greater risk of irinotecan toxicity.

### 11.2 Other drugs metabolized by *UGT1A1*

*UGT1A1* is involved the metabolic pathway of many commonly prescribed drugs, especially those prescribed within cancer and for the treatment of human immunodeficiency virus (HIV‐1) infection. In this regard, CPIC published a guideline for *UGT1A1* and atazanavir in 2015, recommending to consider an alternative agent in *UGT1A1* poor metabolizers particularly where jaundice would be of concern to the patient.^94^ Similarly, the DPWG recommends avoidance of atazanavir in *UGT1A1* poor metabolizers if a suitable alternative is available to reduce to risk of jaundice and drug discontinuation (please refer to the ClinPGx database).^25^ Presently, there is insufficient data to support pre‐emptive testing to guide toxicity management for other drugs which are metabolized by UGT1A1, including the cancer treatments pazopanib, nilotinib, erlotinib and belinostat. For these drugs, increased toxicity or increased risk of developing jaundice has been observed in subjects with reduced UGT1A1 enzyme activity. An updated list of drugs metabolized by UGT1A1 with actionable pharmacogenetic annotations in their label can be found in the ClinPGx database.^95^

Sacituzumab govitecan (SG) is a humanized antibody‐drug conjugate (ADC) that targets the surface glycoprotein trophoblast cell‐surface antigen, Trop2, allowing for the intra‐tumour delivery of SN‐38, the active metabolite of irinotecan.^96^ Targeted delivery of SN‐38 aims to increase efficacy and minimize toxicity. In 2025, a systematic review and meta‐analysis showed that individuals with the UGT1A*28/*28 genotype were at an increased risk of Grade 3 or 4 neutropenia (OR = 2.31), anaemia (OR = 2.69) and diarrhoea (OR = 2.15) compared with wild‐type individuals, with a higher OR for the combined toxicities (OR = 7.0).^97^ This has been recently confirmed in a meta‐analysis of breast cancer studies together with a pharmacokinetic analysis indicating increased exposure to SN‐38 in patients treated with SG in comparison to irinotecan.^98^ The SmPC does not provide guidance for *UGT1A1* poor metabolizers but states that poor metabolizers should be closely monitored.^99^ These meta‐analyses provide evidence for the potential value of pre‐emptive genotyping in patients receiving SG, but whether and how this is implemented will require clinical consultation.

## 12 RESEARCH RECOMMENDATIONS

During development of the guideline, it has become evident that there are evidence gaps. We highlight a non‐exhaustive list of these gaps, which represent key opportunities for further investigation to strengthen the evidence base.

- Prospective, multi‐arm clinical trials evaluating both safety and survival outcomes across a range of genotype‐guided dose reductions are warranted to establish the optimal dose that mitigates toxicity without compromising therapeutic efficacy.
- Further investigation is needed to determine whether irinotecan dose escalation within wild‐type (UGT1A1 *1/*1) or gain‐of‐function *UGT1A1* *36 allele carriers can be safely implemented.
- Real‐world observational data are required on the clinical impact of rare loss‐of‐function *UGT1A1* variants, for example, UGT1A1*7, UGT1A1*27 or UGT1A1*37 alleles.
- Real‐world observational data are also required on the clinical impact of dose titration post cycle 1 dose reductions for poor metabolizers, for example, UGT1A1*28/*28.
- Evidence is required in non‐epithelial paediatric cancers such as Ewing sarcoma or rhabdomyosarcoma where the dose schedules are different from those used in epithelial cancers. In particular, this should evaluate the effect of *UGT1A1* genotyping in this predominantly paediatric population on clinical outcomes (efficacy and safety) with the use of different dosing regimens, with concomitant assessment of pharmacokinetic changes that are seen with the different *UGT1A1* genotypes. The profile of adverse events in the paediatric population should be compared with those in adult populations.
- The effect of concomitant oral cephalosporin, for example, cefixime, treatment to prevent the risk of CTCAE Grade 3–4 irinotecan‐induced diarrhoea needs to be evaluated in terms of clinical outcomes and mechanisms, together with an assessment of any impact on antimicrobial resistance patterns in this patient population.
- There is a need for further studies in other non‐epithelial cancers such as glioblastoma multiforme where irinotecan is used in combination chemotherapy at varying doses. This should also take into account concomitant medications which can affect the expression of UGT1A1, for example, enzyme‐inducing anti‐seizure medications.
- There is a need for studies investigating the interaction between conventional risk factors for toxicity (age, gender, performance status, hepatic impairment or renal impairment) and the impact of supportive treatment, such as GCSF and *UGT1A1* genotype status.
- The overwhelming majority of evidence relating irinotecan‐induced toxicity and UGT1A1 activity is based on Caucasian and East Asian populations. Further real‐world studies determining replicability of these findings across the diverse UK and global populations are warranted.
- A UK‐specific evaluation of the cost‐effectiveness using up‐to‐date costings of genotype‐guided dosing of irinotecan is needed given that the UK‐based economic evaluations were published 14 years ago.

## AUTHOR CONTRIBUTIONS

Dharmisha Chauhan chaired the guideline committee. Dharmisha Chauhan, Cinzia Dello Russo and Munir Pirmohamed contributed to the study conceptualization. Dharmisha Chauhan, Cinzia Dello Russo and Munir Pirmohamed contributed to the study methodology. Jack Thompson, Cinzia Dello Russo and Dharmisha Chauhan contributed to the background and evidence overview. All authors contributed to the recommended indication for pharmacogenetic testing, integration of pharmacogenetic testing into existing clinical pathways and clinical actions based on genotype. Dharmisha Chauhan worked on the summary tables, for example, clinical recommendations by CERSI‐PGx and by other consortia or guidelines. Dharmisha Chauhan, Elisabeth Rolf and Shirley Heggarty inputted on the details of pharmacogenetic testing. Dyfrig A Hughes and Katherine Payne curated the Health Economics section. Cinzia Dello Russo reviewed the evidence related to other pharmacogenetic guidelines. Munir Pirmohamed is responsible for resources and funding acquisition. Dharmisha Chauhan, Cinzia Dello Russo, Jack Thompson, Dyfrig Hughes, Ketherine Payne, Munir Pirmohamed wrote the original draft. All authors contributed to writing review and editing. Dharmisha Chauhan, Cinzia Dello Russo and Munir Pirmohamed revised the guideline according to the feedback received after the consultation process. All authors approved the revised version of the guideline and comments provided in the Supporting Information.

## CONFLICT OF INTEREST STATEMENT

The conflict‐of‐interest statements are documented in Table S2.

## Supporting information


**Table S1:** Examples of irinotecan treatment schedules used within the UK.
**Table S2.** Competence, affiliations, and disclosure of conflicts of interest of the writing committee of the CERSI‐PGx Guideline for *UGT1A1* genotype testing for irinotecan.
**Table S3.** Comments received during the consultation period by various organizations on the CERSI‐PGx Guideline for *UGT1A1* genotype testing for irinotecan and Responses by the UK CERSI‐PGx writing committee.

## Data Availability

The data that support the findings of this guideline are publicly available, as referenced.
