# Supplementary material for: UGT1A1 genotype testing for irinotecan: A guideline developed by the UK Centre of Excellence in Regulatory Science and Innovation in Pharmacogenomics (CERSI‐PGx)
Source: Br J Clin Pharmacol. 2026 Jul 2;92(9):2986–3001. doi: 10.1002/bcp.70659 (PMC13519796; doi:10.1002/bcp.70659)
Supplement: Supplementary file 1 [file BCP-92-2986-s001.docx]

***UGT1A1* genotype testing for irinotecan: A guideline developed by the UK Centre of Excellence in Regulatory Science and Innovation in Pharmacogenomics (CERSI-PGx)**

***Short title:* CERSI-PGx Guideline for *UGT1A1* genotyping with irinotecan**

Dharmisha Chauhan^1,2^, Cinzia Dello Russo^3,4^, Jack Thompson^5^, Dyfrig A Hughes^6^, Katherine Payne^7^, Maria Tsakiroglou^3^, Paul Ross^5^, Rena Chauhan^5^, Simon Jenkinson^8^, Jessica Keen^9,10^, Sophie Harding^11^, Eleanor Lau^12^, Michael Braun^9^, Roshan Agarwal^13^, Richard Adams^14,15^, Elisabeth Rolf^2^, Farah Al-Sheikhli^16^, Sarah Mitchell^9^, Ruth Hammond^17^, Rosie Roberts^14^, Lorna Cairns^18^, Shirley Heggarty^19^, Janet Graham^20^, Justin Mencel^2^, Munir Pirmohamed^3,21^

1. North Thames Genomic Medicine Service Alliance, Great Ormond Street Hospital for Children NHS Foundation Trust, London, UK
2. Royal Marsden NHS Foundation Trust, London UK
3. Department of Pharmacology and Therapeutics, Institute of Systems, Molecular and Integrative Biology University of Liverpool, Liverpool, UK
4. Department of Translational Medicine and Surgery, Section of Pharmacology, Università Cattolica del Sacro Cuore - Fondazione Policlinico Universitario A. Gemelli, IRCCS, Rome, Italy
5. Guy’s and St Thomas’ NHS Foundation Trust, London, UK
6. Centre for Health Economics and Medicines Evaluation, North Wales Medical School Bangor University, Bangor, UK
7. Centre for Health Economics Faculty of Biology, The University of Manchester, Manchester, UK
8. Royal Free London NHS Foundation Trust, London, UK
9. Christie NHS Foundation Trust, Manchester, UK
10. Division of Evolution, Infection and Genomics, University of Manchester, Manchester, UK
11. University Hospital of Wales, Cardiff and Vale University Health Board, UK
12. Swansea Bay University Health Board, Swansea, UK
13. University Hospitals of Northamptonshire, Northampton, UK
14. Velindre Cancer Centre, Velindre University NHS Trust, UK
15. Cardiff University, Cardiff, UK
16. Dorset County Hospital NHS Foundation Trust, Dorchester, Dorset, UK
17. Mount Alvernia Hospital, Guildford, UK
18. Western Health and Social Care Trust, Londonderry, Northern Ireland
19. Belfast Health and Social Care Trust, Belfast, Northern Ireland
20. West of Scotland Cancer Network, Scotland, UK
21. Wolfson Centre for Personalised Medicine, Centre for Drug Safety Science, University of Liverpool, Liverpool, UK

**Supplementary Methodology**

**Writing committee**

The UK CERSI-PGx guideline on *UGT1A1* genotype testing for irinotecan was developed by a multidisciplinary team, including experts in the following disciplines: clinical pharmacology, pharmacology and pharmacogenomics; medical oncology (colorectal cancer, gastrointestinal cancer, cervical cancer; cancer genetics); clinical pharmacy (oncology: lung cancer, gastrointestinal cancer and breast cancer) and pharmacy; clinical nursing in oncology (colorectal cancer), clinical genetics, genomic medicine and health economics. The writing committee included five members from Wales, two members from Northern Ireland and one from Scotland in addition to members from England. Full disclosure of the affiliations, competences and conflict of interests is provided in a separate document in **Supplementary Table 2**. Two preliminary meetings between DC and CDR were held on the 07 and 22 of July 2025 to develop a working plan and the initial draft of the guideline. In addition, JT contributed to the preliminary work, particularly to the literature review for the background overview and the evidence overview sections of the guideline. The writing committee convened the first time online on the 10 October 2025 and subsequently met online twice (on 17 November and 08 December) to finalise the guideline draft for consultation, with written contributions from members throughout this period and after the last meeting. An *ad hoc* meeting was held on the 24 April in response to the issues raised by Paediatric Oncologists and consensus was sought around clinical recommendations for genotyping in patients with non-epithelial cancers.

**Standardised template for the guideline**

The guideline is written according to a standard template developed previously by CERSI-PGx for their guidelines^1^.

**Literature review and prescribing recommendations**

Recommendations from the UK CERSI-PGx consortium are evidence-based together with expert input from committee members who were able to highlight nuances in different oncological settings in which the drug is used. A comprehensive literature review was conducted on the impact of *UGT1A1* genetic variability on irinotecan toxicity as well as efficacy, integrating information provided by the latest clinical pharmacogenetic guidelines developed by the Dutch Pharmacogenetics Working Group^2^. Evidence about clinical feasibility of pharmacogenetic testing was also included. In addition, an extensive literature review was performed in relation to the cost-effectiveness of *UGT1A1* genotyping for irinotecan, prioritising studies relevant to the UK NHS when available. The section on clinical actions based on pharmacogenetic test results contains prescribing recommendations on reducing irinotecan dose to prevent toxicity without affecting efficacy.

**Consultation process**

At the end of the writing process, the guideline final draft was shared both internally (for two weeks) to collect final inputs from members who could not attend online meetings and externally in confidence for consultation (for three weeks). The process involved Regulators (the Medicines and Healthcare products Regulatory Agency, The National Institute for Health and Care Excellence and the National Pharmacogenomics Group (NHS Wales)), Specialty Societies relevant to the prescription of irinotecan, Pharmacy Professional Networks, the ClinPGx (CPIC) and the DPWG. In addition, the guideline was sent out for consultation to the Association of the British Pharmaceutical Industry and The British in vitro Diagnostics Association. The consultation period started on the 9 of March 2026 and was open until the 31 of March 2026. Comments received are included in a single table together with the UK CERSI-PGx responses to each of the comments, and if appropriate, by changes in the guideline. The content of the table is published as **Supplementary Table 3** to ensure transparency of the procedures undertaken.

**Patient And Public Involvement and Engagement (PPIE)**

The guideline was shared with two patient representatives, members of the PPIE group established for the delivery of work package 5 of the UK CERSI-PGx project^3^. One of them participated to the last online meeting held by the writing committee on the 8 of December 2025. A lay summary has been prepared with their contribution to explain the relevance of *UGT1A1* genetic variability on the benefits/risks profile of irinotecan. The lay summary also highlights the impact of genotyping on drug prescription. The lay summary is included in the supplementary material and will be available also through the UK CERSI-PGx website (<https://cersi-pgx.org/>).

**References to the Supplementary Methodology.**

1. Dello Russo C et al. CYP2C19 genotype testing for clopidogrel: A guideline developed by the UK Centre of Excellence in Regulatory Science and Innovation in Pharmacogenomics (CERSI-PGx). *Br J Clin Pharmacol*. 2026; 92: 329-347. doi: 10.1002/bcp.70370.
2. Hulshof EC, Deenen MJ, Nijenhuis M, Soree B, Boer-Veger NJ de, Buunk AM, et al. Dutch pharmacogenetics working group (DPWG) guideline for the gene–drug interaction between UGT1A1 and irinotecan. *European Journal of Human Genetics*. 2023; 31: 982–987. doi: 10.1038/s41431-022-01243-2.
3. Pirmohamed M, Dello Russo C. UK Centre of Excellence in Regulatory Science and Innovation in Pharmacogenomics. *Br J Clin Pharmacol*. 2026; 92(2):327-328. doi: 10.1002/bcp.70363.

**Supplementary Plain English Summary**

Abdur Rehman Hussain and Kamil Sterniczuk, members of the UK CERSI-PGx PPIE group.

**What is Irinotecan?**

Irinotecan is a medicine used in the treatment of a number of cancers, most commonly bowel cancer. It is usually used in combination with other medicines. Irinotecan is given through a drip into a vein, but has also been used orally in children. Once in the body, it is rapidly broken down into SN-38, which is the active substance that slows down or stops the growth of cancer cells. Once SN-38 has worked against the cancer, the body needs to clear it. If SN-38 is not effectively removed, it builds up in the body leading to serious side effects, most commonly a reduced number of white blood cells (which increases the risk of infection) and diarrhoea.

**Why does UGT1A1 matter?**

UGT1A1 is a protein (an enzyme) made in the liver that helps remove SN-38 from the body. The instructions for making this protein come from a gene called *UGT1A1*, which can be inherited in different forms. This means that people clear SN-38 from the body at different speeds. If the *UGT1A1* gene works well, SN-38 is cleared at normal rates. In some people, the enzyme does not work as efficiently, and therefore SN-38 builds up in the body, which increases the risk of the serious side effects mentioned above.

**Different types of response**

People who inherit the most common version of the *UGT1A1* gene are called “normal metabolisers”, and they can clear SN-38 at the expected rate. Some people are called “poor metabolisers” because the removal of SN-38 is reduced by 70%. Other people are called “intermediate metabolisers” because the removal of SN-38 is reduced by 30%.

The guideline recommends that doctors consider a genetic test to determine how well a patient will be able to clear SN-38 from the body when being treated for bowel cancer and other “epithelial” cancers, i.e. cancers which start in the lining of organs. If the patient is a poor metaboliser, the dose of irinotecan should be reduced by 30% to lower the risk of side effects. For normal and intermediate, no change in dosing is required. However, for non-epithelial cancers, for example those arising in muscle, there is inadequate evidence at this stage to recommend genetic testing.

**What this means for patients**

Testing helps doctors give each patient the right dose of irinotecan reducing the risk of the harmful side effects affecting the bone marrow and colon. The results should be recorded in the patient’s medical records so that the safest and most effective treatment plan can be followed. Patients should also be informed why the test is important and what the results mean for their care.

**Regulatory considerations**

Different guidelines from around the world support genetic testing before using irinotecan and recommend a lower dose for people who cannot clear the drug well (poor metabolisers).

**Key message**

Irinotecan is an important medicine used to treat different forms of cancer. A simple genetic test can show whether a patient with an epithelial cancer such as bowel cancer can take the standard dose safely. If not, the dose can be reduced. This approach helps make treatment more personal, lowers the risk of harmful side effects and potentially will also reduce unnecessary interruptions or stopping of treatment.

**Table S1: Examples of irinotecan treatment schedules used within the UK.**

This table is non-exhaustive and does not account for where the treatment lies within specific disease settings, but merely provides some examples of regimens prescribed within the UK.

| **Clinical indication** | **SACT Dosing** | **NHS Acronym** | **Frequency** | **Reference(s)** |
| --- | --- | --- | --- | --- |
| ***Gastrointestinal malignancies*** | | | | |
| Colorectal | Irinotecan 250 to 200mg/m^2^ Day 1  Capecitabine 2000 to 1700mg/m^2^ Day 1 to 14  ***Modified regimen***  Irinotecan 180mg/m^2^ day 1  Capecitabine 1700 to 2000mg/m^2^ Day 1 to 14 | Within the NHS commonly referred to as CAPIRI and XELIRI within clinical studies. | Every 3 weeks | (1–3) |
| Colorectal | *Age < 70 years:*  Irinotecan 350mg/m^2^ Day 1  *Age ≥ 70 years:*  Irinotecan 300mg/m^2^ Day 1 | - | Every 3 weeks | (4,5) |
| Colorectal/pancreatic | Irinotecan 180mg/m^2^ Day 1  5-FU 400mg/m^2^ Day 1  5-FU 2400mg/m^2^ Day 1-2 | FOLFIRI | Every 2 weeks | (6,7) |
| Colorectal/pancreatic | Irinotecan 165mg/m^2^ Day 1  5-FU 3200mg/m^2^ Day 1-2 | FOLFOXIRI | Every 2 weeks | (8) |
| Pancreatic | Irinotecan 180mg/m^2^ Day 1  Oxaliplatin 85mg/m^2^ Day 1  5-FU 2400mg/m^2^ Day 1-2  *Or*  Irinotecan 150mg/m^2^ Day 1  Oxaliplatin 85mg/m^2^ Day 1  5-FU 2400mg/m^2^ Day 1-2 | FOLFIRINOX | Every 2 weeks | (9,10) |
| Gastro-oesophageal | Irinotecan 180 to 150mg/m^2^ Day 1 | - | Every 2 weeks | (11,12) |
| ***Other malignancies*** | | | | |
| Ewings sarcoma – relapsed | Irinotecan 50mg/m^2^ Days 1 to 5  *or*  Irinotecan 10-20 mg/m^2^ Days 1 to 5 and days 8 to 12  In combination with Temozolomide on days 1 to 5 +/- Vincristine | VIT | Every 3 weeks | (13,14) |
| Rhabdomyosarcoma – relapsed | Irinotecan 50 mg/m² on days 1 to 5 in combination with vincristine and temozolomide days 1 to 5 | VIT | Every 3 weeks | (15) |
| Glioma | Irinotecan 125mg/m^2^ on day 1 and 15 |  | Every 4 weeks | (16) |

**References to Table S1.**

1. Fuchs CS, Marshall J, Mitchell E, Wierzbicki R, Ganju V, Jeffery M, et al. Randomized, Controlled Trial of Irinotecan Plus Infusional, Bolus, or Oral Fluoropyrimidines in First-Line Treatment of Metastatic Colorectal Cancer: Results From the BICC-C Study. *Journal of Clinical Oncology*. 2007;25(30): 4779–4786. <https://doi.org/10.1200/jco.2007.11.3357>.

2. Gennatas C, Michalaki V, Gennatas S, Papalambros E. Irinotecan plus capecitabine as first-line chemotherapy in advanced colorectal cancer. *Anticancer research*. 2008;28(3B): 1923–1926.

3. Patt YZ, Lee FC, Liebmann JE, Diamandidis D, Eckhardt SG, Javle M, et al. Capecitabine Plus 3-Weekly Irinotecan (XELIRI Regimen) as First-Line Chemotherapy for Metastatic Colorectal Cancer. *American Journal of Clinical Oncology*. 2007;30(4): 350–357. <https://doi.org/10.1097/coc.0b013e31804b40bb>.

4. Cunningham D, Pyrhönen S, James RD, Punt CJ, Hickish TF, Heikkila R, et al. Randomised trial of irinotecan plus supportive care versus supportive care alone after fluorouracil failure for patients with metastatic colorectal cancer. *The Lancet*. 1998;352(9138): 1413–1418. <https://doi.org/10.1016/s0140-6736(98)02309-5>.

5. Rougier P, Cutsem EV, Bajetta E, Niederle N, Possinger K, Labianca R, et al. Randomised trial of irinotecan versus fluorouracil by continuous infusion after fluorouracil failure in patients with metastatic colorectal cancer. *The Lancet*. 1998;352(9138): 1407–1412. <https://doi.org/10.1016/s0140-6736(98)03085-2>.

6. Citterio C, Baccini M, Orlandi E, Nunzio CD, Cavanna L. Second-line chemotherapy for the treatment of metastatic pancreatic cancer after first-line gemcitabine-based chemotherapy: a network meta-analysis. *Oncotarget*. 2018;9(51): 29801–29809. <https://doi.org/10.18632/oncotarget.25639>.

7. Seymour MT, Maughan TS, Ledermann JA, Topham C, James R, Gwyther SJ, et al. Different strategies of sequential and combination chemotherapy for patients with poor prognosis advanced colorectal cancer (MRC FOCUS): a randomised controlled trial. *The Lancet*. 2007;370(9582): 143–152. <https://doi.org/10.1016/s0140-6736(07)61087-3>.

8. Falcone A, Ricci S, Brunetti I, Pfanner E, Allegrini G, Barbara C, et al. Phase III Trial of Infusional Fluorouracil, Leucovorin, Oxaliplatin, and Irinotecan (FOLFOXIRI) Compared With Infusional Fluorouracil, Leucovorin, and Irinotecan (FOLFIRI) As First-Line Treatment for Metastatic Colorectal Cancer: The Gruppo Oncologico Nord Ovest. *Journal of Clinical Oncology*. 2007;25(13): 1670–1676. <https://doi.org/10.1200/jco.2006.09.0928>.

9. Conroy T, Hammel P, Hebbar M, Abdelghani MB, Wei AC, Raoul JL, et al. FOLFIRINOX or Gemcitabine as Adjuvant Therapy for Pancreatic Cancer. *New England Journal of Medicine*. 2018;379(25): 2395–2406. <https://doi.org/10.1056/nejmoa1809775>.

10. Conroy T, Desseigne F, Ychou M, Bouché O, Guimbaud R, Bécouarn Y, et al. FOLFIRINOX versus Gemcitabine for Metastatic Pancreatic Cancer. *New England Journal of Medicine*. 2011;364(19): 1817–1825. <https://doi.org/10.1056/nejmoa1011923>.

11. Hironaka S, Ueda S, Yasui H, Nishina T, Tsuda M, Tsumura T, et al. Randomized, Open-Label, Phase III Study Comparing Irinotecan With Paclitaxel in Patients With Advanced Gastric Cancer Without Severe Peritoneal Metastasis After Failure of Prior Combination Chemotherapy Using Fluoropyrimidine Plus Platinum: WJOG 4007 Trial. *Journal of Clinical Oncology*. 2013;31(35): 4438–4444. <https://doi.org/10.1200/jco.2012.48.5805>.

12. Kang JH, Lee SI, Lim DH, Park KW, Oh SY, Kwon HC, et al. Salvage Chemotherapy for Pretreated Gastric Cancer: A Randomized Phase III Trial Comparing Chemotherapy Plus Best Supportive Care With Best Supportive Care Alone. *Journal of Clinical Oncology*. 2012;30(13): 1513–1518. <https://doi.org/10.1200/jco.2011.39.4585>.

13. Gupta A, Dietz MS, Riedel RF, Dhir A, Borinstein SC, Isakoff MS, et al. Consensus recommendations for systemic therapies in the management of relapsed Ewing sarcoma: A report from the National Ewing Sarcoma Tumor Board. *Cancer*. 2024;130(23): 4028–4039. <https://doi.org/10.1002/cncr.35537>.

14. Slotkin EK, Meyers PA. Irinotecan dose schedule for the treatment of Ewing sarcoma. *Pediatric Blood & Cancer*. 2023;70(1): e30005. <https://doi.org/10.1002/pbc.30005>.

15. Defachelles AS, Bogart E, Casanova M, Merks JHM, Bisogno G, Calareso G, et al. Randomized Phase II Trial of Vincristine-Irinotecan With or Without Temozolomide, in Children and Adults With Relapsed or Refractory Rhabdomyosarcoma: A European Paediatric Soft Tissue Sarcoma Study Group and Innovative Therapies for Children With Cancer Trial. *Journal of Clinical Oncology*. 2021;39(27): 2979–2990. <https://doi.org/10.1200/jco.21.00124>.

16. Vredenburgh JJ, Desjardins A, Reardon DA, Friedman HS. Experience with irinotecan for the treatment of malignant glioma. *Neuro-Oncology*. 2009;11(1): 80–91. <https://doi.org/10.1215/15228517-2008-075>.

**Table S2.** Competence, affiliations, and disclosure of conflicts of interest of the writing committee of the CERSI-PGx Guideline for *UGT1A1* genotype testing for irinotecan.

| **Name** | **Main Expertise** | **Full Affiliation** | **Institution/s** | **Conflict of Interest** | **Nation** |
| --- | --- | --- | --- | --- | --- |
| Dharmisha Chauhan CHAIR | Genomic Medicine  Oncology Pharmacy, Lung Cancer | North Thames Genomic Medicine Service Alliance  Royal Marsden NHS Foundation Trust | North Thames Genomic Medicine Service Alliance  Royal Marsden NHS Foundation Trust | Speaker honoraria for educational content: AstraZeneca, Bristol-Myers Squibb, Merck, MSD and Boehringer Ingelheim  Consultancy: Boehringer Ingelheim | England |
| Munir Pirmohamed | Clinical Pharmacology Internal Medicine  Pharmacogenomics | Department of Pharmacology and Therapeutics, Wolfson Centre for Personalised Medicine, University of Liverpool, Liverpool, UK.  Liverpool University Hospital Foundation NHS Trust, Liverpool, UK. | The University of Liverpool | Currently receives partnership funding, paid to the University of Liverpool, for the MRC Medicines Development Fellowship Scheme (co-funded by MRC and GSK, AZ, Optum and Hammersmith Medicines Research). He has developed an HLA genotyping panel with MC Diagnostics but does not benefit financially from this. He is part of the IMI Consortium ARDAT ([www.ardat.org](http://www.ardat.org)); none of these of funding sources have been used for the guideline | England |
| Cinzia Dello Russo | Pharmacology  Pharmacogenomics | Department of Pharmacology and Therapeutics, Institute of Systems Molecular and Integrative Biology (ISMIB), University of Liverpool, Liverpool, UK.  Department of Translational Medicine and Surgery, Section of Pharmacology, Università Cattolica del Sacro Cuore - Fondazione Policlinico Universitario A. Gemelli, IRCCS, Rome, Italy | The University of Liverpool  Università Cattolica del Sacro Cuore - Fondazione Policlinico Universitario A. Gemelli, IRCCS, Rome, Italy. | No conflicts | England |
| Rena Chauhan | GI cancer pharmacy, Non-medical prescriber | Guy's And St Thomas' NHS Foundation Trust | Guy's And St Thomas' NHS Foundation Trust | No conflicts | England |
| Simon Jenkinson | GI and Breast cancer pharmacy  Non-medical prescriber | Royal Free London NHS Foundation Trust | Royal Free London NHS Foundation Trust  England, London | No conflicts | England |
| Farah Al-Sheikhli | Pharmacy, GI Cancer | Dorset County Hospital NHS Foundation Trust | Dorset County Hospital NHS Foundation Trust | Has received educational sponsorship from Amgen, Pfizer, and Chugai Pharma to support attendance at professional conferences.  Involvement in national groups (UKAOS, BOPA AI Committee, BOPA Digital Committee, BMT Pharmacy Committee, UKMS Pharmacy Working Group). | England |
| Jessica Keen | Genomic Medicine  GI cancer pharmacist | Division of Evolution, Infection and Genomics, University of Manchester  The Christie NHS Foundation Trust | University of Manchester  The Christie NHS Foundation Trust | Currently receiving fellowship funding from the Wellcome 4Ward North PhD academy. Funding source was not used in guideline development. | England |
| Sophie Harding | Pharmacy  Genomic medicine  Oncology | Consultant Pharmacist for Genomics & Pharmacogenomics  Based at: University Hospital of Wales | University Hospital of Wales | No conflicts declared | Wales |
| Eleanor Lau | Pharmacy, colorectal cancers | Swansea Bay UHB | Swansea Bay UHB | Honoraria from Amgen, Merck, Servier and Bayer | Wales |
| Michael Braun | Oncology, colorectal cancers<mailto:michael.braun@nhs.net> | Christie NHS Foundation Trust | Christie NHS Foundation Trust | Received educational grants or support for conference attendance from the following companies – Servier, Pierre-Fabre, BMS, Merck, Amgen and Takeda. | England |
| Roshan Agarwal | Oncology, bowel and cervical cancers | University Hospitals of Northamptonshire | University Hospitals of Northamptonshire | No conflicts declared | England |
| Paul Ross | Oncology, GI cancer | Guy’s and St Thomas’ NHS Foundation Trust, London | Guy’s and St Thomas’ NHS Foundation Trust, London | No conflicts declared | England |
| Richard Adams | Oncology | Cardiff University and Velindre Cancer Centre | Cardiff University and Velindre Cancer Centre | No conflicts declared | Wales |
| Justin Mencel | Cancer genetics  Colorectal oncology | The Royal Marsden NHS Foundation Trust | The Royal Marsden NHS Foundation Trust | Speaker honoraria: Bristol-Myers Squibb, Tempus, Servier  Consulting/Advisory board: Daiichi, Astra-Zeneca, Jazz  Travel support: Astra-Zeneca, BeOne | England |
| Elisabeth Rolf | Clinical Scientist  Cancer genomics | The Royal Marsden NHS Foundation Trust | The Royal Marsden NHS Foundation Trust | No conflicts | England |
| Sarah Mitchell | Nursing, colorectal cancers | The Christie NHS Foundation Trust  Manchester | The Christie NHS Foundation Trust Manchester | No conflicts | England |
| Ruth Hammond | Nursing, oncology  (member of SACT Board and UKONS) | Clinical Service Manager  Mount Alvernia Hospital  Harvey Road  Guildford, GU1 3LX | UKONS Board Member and SACT Committee member | No conflicts | England |
| Rosie Roberts | Nursing, Oncology  (SACT Board Member) | Nurse Consultant SACT,  Velindre Cancer Centre, Cardiff  & Lead SACT Nurse, NHS Wales National Cancer Team | Velindre Cancer Centre,  Wales & NHS Wales  National Cancer Team | No conflicts | Wales |
| Jack Thompson | Clinical Pharmacology | Guy’s and St Thomas’ NHS Foundation Trust, London | Guy’s and St Thomas’ NHS Foundation Trust, London | No conflicts | England |
| Maria Tsakiroglou | Clinical Pharmacology | Institute of Systems, Molecular and Integrative Biology (ISMIB), Department of Pharmacology and Therapeutics, University of Liverpool | The University of Liverpool | No conflicts | England |
| Katherine Payne | Health Economics | Manchester Centre for Health Economics Faculty of Biology, Medicine and Health , 4th floor, Jean McFarlane Building The University of Manchester | The University of Mancheste**r** | No conflicts | England |
| Dyfrig Hughes | Health Economics  Pharmacy  Pharmacogenomics | Co-Director, Centre for Health Economics & Medicines Evaluation,  North Wales Medical School | Bangor University | Chair of NHS Wales National Pharmacogenomics Group, and co-chair of NHS England Pharmacogenomics Test Evaluation Working Group | Wales |
| Lorna Cairns | Pharmacy | Western Health and Social Care Trust  Northern Ireland | Western Health and Social Care Trust  Northern Ireland | No conflicts declared | Northern Ireland |
| Shirley Heggarty | Clinical Genetics | Belfast Health and Social Care Trust  Belfast | Belfast Health and Social Care Trust  Belfast  Northern Ireland | No conflicts declared | Northern Ireland |
| Janet Graham | Medical Oncology | West of Scotland Lower GI Managed Clinical Network | West of Scotland Lower GI Managed Clinical Network | Consultancy fees or honoraria GSK, Astellas, Takeda, Merck Serono and Merck Sharp & Dohm, MJH Healthcare Holdings, Merck Health Care, Servier Laboratories, BMS | Scotland |

**Table S3.** Comments received during the consultation period by various organizations on the CERSI-PGx Guideline for *UGT1A1* genotype testing for irinotecan and Responses by the UK CERSI-PGx writing committee.

| **Organisation** | **Comments from organisation** | **Responses from Committee** | **Changes to the Guideline (if any)** |
| --- | --- | --- | --- |
| MHRA | **Page 1** – but also in other part of the paper where there is reference to indications:  *Irinotecan is a topoisomerase I inhibitor used as a first line treatment option for advanced colorectal and pancreatic cancer in combination with 5-fluorouracil (5-FU). It is also considered as monotherapy in recurrent oesophageal or gastric cancer, or as combination treatment for progressive Ewing sarcoma and rhabdomyosarcoma.*  ***MHRA comment***  - The paragraph above may imply to the reader that irinotecan is authorised for treatment of colorectal cancer and pancreatic cancer.  It is important to distinguish between non-liposomal/non-pegylated irinotecan and “Onivyde pegylated liposomal” which is the pegylated liposomal irinotecan and their different authorised indications.  As per the SmPC: “*ONIVYDE pegylated liposomal is not equivalent to non-liposomal irinotecan formulations and should not be interchanged”.*  Irinotecan is currently authorised for the treatment of colorectal cancer (see SmPC for full details). However, “*ONIVYDE pegylated liposomal”* is currently authorised for the treatment of pancreatic cancer (see SmPC for full details).  - In addition, throughout the paper, the authors have referred to indications which appear to be off-label use of irinotecan. The authors may consider reviewing these.  The reader may perceive the off-label indications as an endorsement by the authors of this guideline.  **Page 3**  *UGT1A1*28 has an additional dinucleotide (thymine-adenine) tandem repeat within the TATA box…*  ***MHRA comment***  On page 3, there is reference to “TATA” but the reader may not be familiar with this abbreviation. It is assumed that the authors refer to thymine–adenine (TA). If so, the authors may consider providing the full wording for this abbreviation.  Based on Dean et al’s paper, “*UGT1A1*28* variant contains an extra thymine–adenine (TA) repeat within the TATA promoter region, 7 TA repeats compared with 6 in the wild type allele…”. The authors may consider providing context with regards to the wild-type and expand this section as appropriate.  **Page 6**  *Irinotecan is available in the UK as conventional (CAMPTO® and generics) and liposomal (ONIVYDE® pegylated liposomal) formulations for intravenous infusion. These formulations are not interchangeable. Pegylated liposomal irinotecan is licensed but has not been recommended for use in the National Health Service (NHS) by the National Institute for Health and Care Excellence (NICE) (25). ...*    *Pegylated liposomal irinotecan is indicated:*   - *in combination with oxaliplatin, 5-fluorouracil (5-FU) and leucovorin (LV) for the first-line treatment of adult patients with metastatic adenocarcinoma of the pancreas,* - *in combination with 5-FU and LV for the treatment of metastatic adenocarcinoma in adult patients who have progressed following gemcitabine-based therapy.*   ***MHRA comment***  Name of the products:  It is noted that the authors refer to “*Pegylated liposomal irinotecan is indicated…”*  - The use of the accurate name of the medicinal product as per SmPC is important as it may lead to confusion and potential for medication error. The authors are requested to use the naming as per the SmPC/naming convention i.e. name pegylated liposomal i.e. Onivyde pegylated liposomal and with explanatory notes that Onivyde pegylated liposomal contains the active substance irinotecan. However, Onivyde pegylated liposomal is not equivalent to non-liposomal irinotecan formulations and should not be interchanged.  Please also ensure using a consistent product name throughout the document. i.e. Onivyde pegylated liposomal  **Page 7**  *It is important to note that the indications for irinotecan have been expanded within the NHS beyond the approved therapeutic indications listed above, with irinotecan prescribed from neoadjuvant through to advance disease clinical settings.* ***Table 3*** *provides some examples of clinical areas which routinely consider the use of irinotecan…*  ***MHRA comment***  As per previous comment, the above statement and table 3, may be perceived as endorsement by the authors of this guideline. The authors may consider reviewing and revising this section as appropriate.  **Page 21**  **10. REGULATORY CONSIDERATIONS**  The UK SmPC describes the role of *UGT1A1* variants increasing the risk of severe diarrhoea and neutropenia, especially at increased irinotecan dose levels, stated as >180mg/m^2^ within section 4.4 (26). Within section 5.2, reference is made to the well characterised variants *UGT1A1*28* and *UGT1A1*6* and acknowledges the data associating an increased risk of neutropenia and diarrhoea in homozygote carriers, but no guidance is provided on recommended starting doses for poor metabolisers (26). In contrast, the SmPC (section 4.2) for pegylated liposomal irinotecan provides clear dose reduction guidance for *UGT1A1* poor metabolisers stating a starting dose of 50mg/m^2^, a 30% dose reduction from the licensed 70 mg/m^2^ dosing (27). This guidance is based on the NAPOLI-1 randomised controlled trial where *UGT1A1*28/*28* homozygote carriers were prescribed an upfront irinotecan dose reduction of 50mg/m^2^ (27). There is no other published UK based guidance outside of the SmPCs.  ***MHRA comment***  With respect to *“The UK SmPC describes the..”* Please clarity which SmPC is being referred to ..  ***MHRA comment***  **Gilbert’s syndrome**: The guideline refers to Gilbert’s syndrome and that “*UGT1A1* loss of function alleles have been clinically associated with Gilbert’s syndrome” but it doesn’t explicitly say that patients with Gilbert’s syndrome may be at increased risk of toxicity with irinotecan. In particular given that Gilbert’s syndrome is a common condition which this paper acknowledges. As per [NICE CKS](https://cks.nice.org.uk/topics/gilberts-syndrome/background-information/prevalence/) in western Europe, 5–10% of the population are affected, the prevalence of Gilbert's syndrome differs between ethnic groups and is up to approximately 20% in South Asian and some Middle Eastern (Iranian) populations….    The [SmPC for Campto](https://www.medicines.org.uk/emc/product/9083/smpc) (irinotecan) ***Patients with reduced UGT1A1 activity*** *Patients that are UGT1A1 poor metabolisers, such as patients with Gilbert's syndrome (e.g. homozygous for UGT1A1*28 or *6 variants) are at increased risk for severe neutropenia and diarrhoea following irinotecan treatment. This risk increases with the irinotecan* dose *level.*  The SmPC for [ONIVYDE pegylated liposomal 4.3 mg/ml concentrate for dispersion for infusion - Summary of Product Characteristics (SmPC) - (emc) \| 9200](https://www.medicines.org.uk/emc/product/9200/smpc)  *Patients with deficient glucuronidation of bilirubin, such as those with Gilbert's syndrome, may be at greater risk of myelosuppression when receiving therapy with ONIVYDE pegylated liposomal.*    **Other editorial comments:**   - **page 7*- 2.1 Expansion of irinotecan indications*** *It is important to note that the indications for irinotecan have been expanded within the NHS beyond the approved therapeutic indications listed above,……………*  please consider revising this section - **Page 13 -** *UGT1A1* should be requested prior to the treatment of an irinotecan-based regimen …………. Please consider wording – is it intended to be UGT1A1 testing? - **Page 14**   *are* found within the TATA section of the 5' promotor – please review as “are” is in italics” and please review spelling of “promotor” - **Page 15- edit:** please consider removing for; **….***. This timepoint is from the time the sample is received by the laboratory to the results being returned for to the clinician for clinical action prior to treatment administration…* - **Page 17-**  please review Spelling of hematological | We thank the MHRA for this comment and amended the guideline accordingly to clarify the different irinotecan formulations. Where needed, the brand name for the irinotecan products is described. In **section 2**, we have highlighted which indications are licensed and where current usage is off-label. We have also clarified that where irinotecan is mentioned this relates to the non-pegylated formulation only (**Section 2.1**).  As suggested, we provided greater explanation about TA repeats.  Thank you for this comment. Amendments were made to ensure that the brand name ONIVYDE is now included where the pegylated liposomal formulation has been mentioned.  We thank the MHRA for this comment. We have now clearly stated that the indications reported in Section 2.1 and in Table 3 are off-label uses of irinotecan. We included these clinical uses of the drug (conventional formulation), since they reflect current practice in the UK.  As suggested, we clarified which SmPC we are referring to.  Thanks for your comment, we have now included the potential increased risk of irinotecan toxicity in subjects with Gilbert’s syndrome.  All editorial changes highlighted have been amended. | Yes – Abstract, Section 2 and Section 10.  Yes – Section 1  Yes - Section 2  Yes - Section 2.1  Yes – Section 10  Yes – Section 11.1  Yes - Section 2.1, Section 5, Section 6, Section 8.4 |
| NICE | No response received |  |  |
| British Pharmacological Society | No response received |  |  |
| UK Clinical Pharmacy Association | We very much welcome this guidance. This is a clear and informative guideline, that is easy to read and interpret. The recommendations for further research are well considered and pragmatic.The UKCPA Genomics committee had a few comments and suggestions for clarifications as follows:    ***3.2 UGT1A1* genotyping and clinical efficacy**  The p values for median OS for the studies conducted by Satake and Ly both show non-significance. However on pg 10, the guideline states *‘ Lv et al. (2024) treated 107 Chinese gastric cancer patients with genotype-based dosing with no differences in outcome (45) while Satake et al. (2022) reported a median overall survival (OS) of 8.8 months in non-poor metabolisers compared to 11.5 months in poor metabolisers (p=0.579)’ .* If no statistically significant difference in outcome in either study was reported, should this not be presented in the same way in the text?  In relation to the AXEPT III study survival rates, the guideline states *‘This difference was attributed to patient’s performance status or disease staging and not to receiving a lower starting dose’.* It is difficult to find this information within the reference itself. In table 1 of the reference, no statistically significant difference in ECOG performance status or number of metastatic sites is reported between UGT1A1 diplotypes.  The authors state that any potential differences in right sided disease presentation between groups were not statistically assessed due to duplicate aggregation.  p.10 ‘*In a Japanese study, Satake et al. retrospectively analysed the clinical outcomes of 235 UGT1A1-genotyped patients with advanced pancreatic cancer receiving FOLFIRINOX  (44) . UGT1A1 poor metabolisers (*6/*6 and *28/*28) started with a lower irinotecan dose, reduced from 150 to 80 mg/m² every two weeks  (44).. Among the 11 poor metaboliser patients, only one developed CTCAE grade ≥3 toxicity, suggesting that larger dose reductions may be required  (44)’’  -* Only one patient from 11 poor metabolisers experienced grade toxicity, which perhaps doesn’t conclusively suggest that the dose reduction failed to prevent toxicity? The separation of discussion of clinical efficacy into section 3.2 makes it trickier to consider data from each study in the round.  A typographical error is noted on p.10 *‘vincristine), every 3 weeks, with weekly cumulative doses ranging between 50 to 250mgmg/m^2^  (48,49)*  P10 “Early studies suggested reduced SN-38 glucuronidation...” This may benefit from re-wording – we are presuming glucuronidation = UGT1A1 activity to break irinotecan down to inactive metabolites?    **6. WHICH GENE(S), VARIANTS, TURNAROUND TIME?**  p.15 - “*For treatments containing 5-FU or its derivatives, clinicians should wait for both the UGT1A1 and DPYD test results.”*  The committee strongly agrees with this statement. Could these possibly be requested under a single test code, where both are indicated, to streamline the pathway?      **11.2 Other drugs metabolised by UGT1A1**  p.22 “*UGT1A1 is involved the metabolic pathway of many commonly prescribed drugs, especially those prescribed within cancer and for the treatment of human immunodeficiency virus (HIV-1) infection. Presently, there is insufficient data to support pre-emptive testing to guide toxicity management for the following drugs which are metabolized by UGT1A1: pazopanib, nilotinib, atazanavir, dolutegravir, and raltegravir.*  “ This aspect should be considered for patient counselling – what information will be provided to patients?    **8. OTHER PHARMACOGENETIC GUIDELINES**  Pg 18 – should it be clarified that the rating ‘A’ from CPIC is provisional, as CPIC guidelines are yet to be developed for this?    **9. HEALTH ECONOMIC EVALUATION**  It would be helpful to acknowledge that the health economic analyses were conducted some time ago (up to 18 years ago) and that the most recent UK-based analysis was conducted 14 years ago.  It is of relevance, given that sequencing and other healthcare/drug costs may have changed since then. | We thank the UK Clinical Pharmacy Association for the positive comments and the support for the development of the UK CERSI-PGx guidelines  The text has been amended according to your suggestion.  The text has been amended according to your suggestion.  Thank you for your comment. Amendment was made to the highlighted sentence in Section 3.1  The typographical error has been corrected.  We rephrased this sentence to reflect your suggestion.  We appreciate your support of our recommendation and thank you for your suggestion. This is certainly an option that would facilitate the access to both tests and should be considered by the laboratories providing the test as well as the NHS  Thank you for your comment which gave us the opportunity to revise this section. We have now included the reference to the CPIC guideline, published in 2015, to optimise the clinical use of atazanavir based on UGT1A1 metabolic status. We listed only cancer treatments for which actionable PGx annotations are included in the drug labels by different Regulatory Agencies as reported in the ClinPGx database. We referred the readers to the relevant ClinPGx section, to facilitate access to updated information of pharmacogenetic annotations related to UGT1A1 in drug labels.  CPIC is currently developing UGT1A1 guidelines with the CPIC level listed as A based on current information on the ClinPGx site. Amended to reflect the provisional status whilst guidance under development.  We agree with your comment and have acknowledged the need for additional health economic analyses, possibly relevant to the UK. | Yes – Section 3.2  Yes – Section 3.2  Yes – Section 3.1  Yes – Section 3.1  Yes – Section 3.2  No  Yes – Section 11.2  Yes - Section 8  Section 12 |
| British Oncology Pharmacy Association (BOPA) | This was discussed at Governance, Advocacy and Publications Subcommittee and happy to endorse. Great piece of work. | We thank the British Oncology Pharmacy Association for the support. | No |
| Paediatric Oncology Pharmacists (POP)(within BOPA) | Comments from POP:   - No paediatric pharmacy and medical representation on the working group - What is the turnaround time for testing? Often these regimens need to start urgently (relapse treatment) - The data presented is in adults and whilst it acknowledges the need for studies in children, we are looking for curative intent and would need to know the impact on clearance and PKs in children. So, we would support in TYA but cannot support the recommendation in point 4 *UGT1A1* testing should be offered to all adult and **paediatric patients** who are due to receive irinotecan-based regimens, irrespective of their ancestry for children. - Could it be that clinicians can request for paediatrics if there are concerns around toxicity? | - Thank you for your comment. We addressed this point by organizing an ad hoc meeting with specialists in paediatric oncology (see bullet points below) - The turnaround time has been described within section 6. - Based on the comments obtained from the external consultation, a separate meeting with sarcoma medical oncology consultants working within paediatric and teenage and young adults was arranged to address the role of UGT1A1 testing within the paediatric population. As a result of this, we have made changes to the recommendations, and to the text within the guideline highlighting the lack of evidence and the need for further research. - This is a very interesting point. We have made this point. | Yes section 3.1, 4 and 12  No  Yes, sections 3.1, 4 and 12  Yes, Section 4 |
| British Sarcoma Group | I have reviewed these and am happy from a sarcoma point of view.  Turnaround time of 3- 5 days for testing sounds good in the context of Ewings and rhabdomyosarcoma | We are thankful to the British Sarcoma Group for the support and the positive feedback on the UK CERSI-PGx guideline for irinotecan. | No |
| UK SACT Board | I know some of the aspects raised below have been covered in the working meetings, however if this is not coming across in the document then maybe it should be more specific re advice on operationalising guidance from a practical standpoint re operational advice in a one-page summary. E.g.,   - For labs: minimum TAT based existing clinical pathways - For nurses: example local blood request form and patient focused explanation narrative (consider patient info leaflet in appendix) - For pharmacists: example protocol wording - For commissioners: risk of not commissioning for this indication - For clinicians: Pathway point. To request via local blood forms. To use clinical judgement if result delayed result re: treatment needs to commence verses delay.   Also is the next step to approach NICE....  Below feedback from members of the **UK SACT**:  I’m very much in favour of working out how we can adopt pharmacogenomic pathways/investigations and data into clinical practice from a SACT safety perspective. We have been talking about UGT1A1 testing for a long time so to adopt this as a standard practice would be a big stride forward.  At present patients are not tested as standard. Because the test is commissioned in Gilbert’s, some clinicians who have patients with raised, unexplained, baseline bilirubin, are checking the genotype prior to irinotecan use under the Gilbert testing commissioned pathway. However, most won’t and due to the indications for irinotecan many patients will have raised bilirubin secondary to malignant so an alternate cause may not be considered.  I am also conscious that there is a risk of perceived safety and delay in diagnosis of drug toxicity in certain cases. Due to the extensive nature of pharmcogenomics, breadth and variable functionality of mutations lack of identification of a mutation runs the risk of being falsely reassuring so the communication around this is key and could be more extensively outlined in the guideline.  In the case of DPYD the commissioned test was only to test four (of at least 15 known) genotypes with associated prescribing recommendations. Despite this only including the four common mutations clinicians on the ground have in some cases interpreted this as normal if none of the variants found and therefore there were cases of delay in diagnosis of 5FU related toxicity as their DPYD test was ‘normal’ and falsely reassuring.  The pharmcogenomic status of UGT1A1 genotype testing could be considered to be more complex than DPYD so although the paper references the potential test outcomes and interpretations I think this does need to be clearly caveated than it is not exhaustive etc.  Looking at the paper there is a health economics section but I don’t think clear if testing UGT1A1 is commissioned for this indication - apologies if I have missed it. I am aware testing is part of the national genomics directory for Gilbert’s syndrome, commissioned in the UK under code R176.1. If it is now commissioned for irinotecan treatment it would be useful to highlight the pathway, code and document to use. If not then in suggesting this recommendation trusts will need to do an analysis to determine cost impact and how to support its introduction in terms of numbers of patients potentially tested and the cost.  Also the NHS list price would be important to know as would whether there is a testing tariff (actually taking the blood) associated with it or if this falls under block.  If there isn’t commissioning pathways it makes it very difficult to implement unless the suggestion is that each trust makes an ICB application (as all commissioning including historic spec comm commission will soon fall under ICBs)? Otherwise there is a guideline to safeguard patients which is not supported in implementation and therefore leaves trusts in a difficult position.  Overall the document is very detailed which is very helpful but would benefit from a clear diagrammatic figure to outline the pathway/ process/ testing outcomes and implementations including turnaround times and how to navigate potential delays. The pathway for DPYD testing is now slick as it has been considered in terms of when to send and optimised turnarounds but unlike 5FU, irinotecan is used in different indications/ lines of treatment in a number of cases so the pathway may well be different despite an overlap in oncology specialisms.  I think adoption of these tests and the potential avoidance/ reduction of toxicities for affected patients is key but would be useful to have the above addressed in the guideline to support clinical teams to use it effectively.  If further review is helpful I would be happy to support. | These comments relate to implementation and deployment within the NHS which is outside the remit of the CERSI-PGx guidelines. However, please note that the TAT was discussed and has been described within Section 6. We acknowledge receipt of the other comments and willing to provide the NHS and genomic medicines services support for protocols on how to request the test and which is the appropriate type of sample if needed.  NICE has been invited to respond to this guidelines during the consultation process.  Thank you for your positive and supportive comment  Thank you for highlighting the need of a specific pathway within the NHS to request the UGT1A1 pharmacogenetic test to optimise the prescription of irinotecan.  Thank you for highlighting the limitation of pharmacogenetic tests based on a limited number of variants and the complexity of *UGT1A1* pharmacogenetic testing, in these three comments. We have included at the end of Section 6 the following sentence: ‘*Clinicians should consider that both tests are designed to identify a limited number of the most common allelic variants therefore reduced enzymatic activity due to rare variants may not be detected. This represents a known limitation of pharmacogenetic tests based on selected variants*’. We hope that this will raise awareness on the meaning of a negative result. We are also conscious that the genomic capability of the NHS is fast changing, so we expect that in the future pharmacogenomic information may be extracted from whole genome sequencing data and this risk would be mitigated.  The Health Economic section aims to provide a concise literature review of the current available health economic analyses on the cost-effectiveness of pharmacogenetic testing considered by the CERSI-PGx guidelines. We are aware of the lack of updated Health economic evaluations relevant to the UK, thus have acknowledged the need for additional health economic analyses, possibly relevant to the the UK.  Thank you for this comment. We are aware that the test for UGT1A1 and irinotecan is not commissioned yet. A request has been made to include this test in the National Genomic Test Directory and we expect that once commissioned clinicians can use it and apply the prescribing recommendations included in the present document.  Relevant information for clinicians is summarized in the abstract section. A diagrammatic figure has not been provided as testing methods and pathways can change over time within the NHS. This guideline provides recommendations to support an ideal testing pathway especially where existing pathways exist, as provided within section 5. We have also stipulated that ideally the request of the PGx test for irinotecan should follow the current NHS DPYD pathway with minimal disruption to current clinical practice.  Thank you for your final comment and support. As mentioned above, once the test for UGT1A1 will be commissioned in the NHS clinicians can fully adopt the UK CERSI-PGx guideline recommendations for the optimal use of the test results. | No  No  No  No  Yes – Section 6  Yes – Section 12  No  No  No |
| UKONS  UK Oncology Nursing | No response received |  |  |
| Royal Pharmaceutical Society | No response received |  |  |
| National Pharmacogenomics Group (NHS Wales) | No response received |  |  |
| ABPI | No response received |  |  |
| BIVDA | No response received |  |  |
| Clin PGx | No response received |  |  |
| DWPG | Below our suggestions:   - In the background and overview section, the wording is very ambiguous regarding minor allele frequency vs. the number of carriers (heterozygous and homozygous) in a population. Phrasing this more clearly would strengthen clinical interpretation, as the average doctor currently reads that, for example, 32-36% of Europeans have *28, whereas I believe that refers to MAF. - On page 8, the first paragraph states that more research into dose escalations in NMs is needed; we agree with this, but think it should be mentioned in this text that a recent, large phase II study by Sanoff et al. was negative and found no added value in higher dosages for NMs (PMID: 38837045). - Section 3.1, at the top of page 10: *“among the 11 PMs, only one developed severe toxicity, suggesting larger dose reductions may be required”* --> this conclusion seems strange: given such a toxicity prevalence (1:11), one could say irinotecan is tolerated quite well? - In sections 3.1 and 3.2, the total numbers per study are discussed, but it would be clearer for the uninformed reader if the numbers of PMs and IM/NMs were mentioned to give an idea ofthe group size.   Finally:   - In Table 4 - overview of survival studies - an important study is missing, being: Peeters et al (PMID: 41777427). This is a bit strange since the paper it is being discussed extensively in the text. - We would suggest to make an explicit recommendation that for patients who need to start irinotecan quickly, and for whom UGT1A1 status is not yet known, the first cycle should be started with a 30% reduction (and thus the patient treated as if they were a PM); for an IM/NM genotype, the dosage can then be increased to 100% in cycle 2. This prevents PMs from accidentally receiving a 100% dosage and subsequently developing severe or even fatal toxicity. | Thank you for your comment. We have now specified that the MAF is reported in this paragraph and in Table 2. We included the phenotype frequencies in Table 7.  Thank you for your comment. We have now included the results from this study in Section 3  Thank you for your comment, we amended the text accordingly.  Thank you for your comment, we amended the text accordingly  Thank you for your comment, we amended Table 4 accordingly.  Thank you for your comment. We have included a statement in the scenario where UGT1A1 status is not yet known to make it an optional recommendation and included a reference. We have also included specific recommendations about pharmacogenetic testing in non-epithelial cancers (section 4) and underlined that the clinical actions based on pharmacogenetic testing refer only to epithelial cancers (section 7), based on input from paediatric oncologists (see next point). | Yes – Section 1  Yes – Section 3  Yes - Section 3.1  Yes - Sections 3.1 and 3.2  Yes - Table 4  Yes – Section 7 |
| Online meeting with representatives (paediatric oncologists)  from the following associations:  CCLG (The Children’s Cancer and Leukaemia Group);  UK3C Sarcoma Group; and EuroEwing Consortium | We have read with interest the CERSI-PGx guideline on the use of UGT1A1 pharmacogenetic testing to prevent irinotecan toxicity. However, concerns arise regarding the recommendation of a 30% dose reduction of irinotecan at cycle 1 in UGT1A1 poor metabolisers in the paediatric setting, where irinotecan is used in combination with other agents for the treatment of various cancers, particularly relapsed tumours such as Ewings sarcoma, rhabdomyosarcoma, and neuroblastoma. The evidence in Ewing sarcoma is to use a daily dose of 10-20mg/m^2^ for either one or two weeks, that is 5 or 10 days. However, in recurrent tumours a high dose of 40-50mg/m^2^ for days 1 to 5, so up to 250mg/m^2^ per cycle, is often empirically used. The schedule of treatment differs from the adults in whom high dose boluses are usually administered. In adult carcinoma, irinotecan is more commonly given at ≥125mg/m^2^ on day 1 and day 8. Therefore, higher C_max_  are reached in adults which can explain the higher toxicity observed in these patients compared to children. In paediatrics, diarrhoea is mostly mild and does not require dose reduction, it is commonly managed empirically. Bone marrow suppression is rarely observed in children with irinotecan/temozolomide regimens. In these patients we may use other agents with a higher immunosuppressant potential than irinotecan/temozolomide, like for example topotecan/cyclophosphamide or ifosfamide (Gerrand C, et al. UK guidelines for the management of bone sarcomas. Br J Cancer. 2025;132: 32-48). These drugs can be used in the paediatric patients with sarcoma as well as neuroblastoma. The UGT1A1 screening test is useful and often requested to identify patients with Gilbert’s syndrome, when hyperbilirubinemia is detected during chemotherapy.  Pharmacokinetic studies of irinotecan in the paediatric setting are limited, particularly for the low-dose, protracted regimens commonly used. There is a need to determine whether C_max_ or overall exposure correlates with efficacy. Therefore, pharmacokinetic studies are essential to ensure that a 30% dose reduction at cycle 1 in UGT1A1 poor metabolisers achieves comparable drug exposure and, consequently, similar efficacy. There is a need for additional studies proving that it is safe to reduce the dose, avoiding toxicity, particularly the diarrhoea, and maintaining as good outcomes as the full dose. | We understand your concerns and we have made changes to the recommendations, and to the text within the guideline highlighting the lack of evidence and the need for further research. | Yes, sections 3.1, 4 and 12 |
